# Industrial water resources management based on violation risk analysis of the total allowable target on wastewater discharge

**DOI:** 10.1038/s41598-017-04508-9

**Published:** 2017-07-11

**Authors:** Wencong Yue, Yanpeng Cai, Linyu Xu, Zhifeng Yang, Xin’An Yin, Meirong Su

**Affiliations:** 1 Research Center for Eco-environmental Engineering, Dongguan University of Technology, Dongguan, 523808 China; 2 State Key Laboratory of Water Environment Simulation, School of Environment, Beijing Normal University, Beijing, 100875 China; 3 School of Environment and Energy, South China University of Technology, Guangzhou, Guangdong, 510006 China; 4 Institute for Energy, Environment and Sustainable Communities, University of Regina, Regina, Saskatchewan, S4S 7H9 Canada; 5 Beijing Engineering Research Center for Watershed Environmental Restoration & Integrated Ecological Regulation, School of Environment, Beijing Normal University, Beijing, 100875 China

## Abstract

To improve the capabilities of conventional methodologies in facilitating industrial water allocation under uncertain conditions, an integrated approach was developed through the combination of operational research, uncertainty analysis, and violation risk analysis methods. The developed approach can (a) address complexities of industrial water resources management (IWRM) systems, (b) facilitate reflections of multiple uncertainties and risks of the system and incorporate them into a general optimization framework, and (c) manage robust actions for industrial productions in consideration of water supply capacity and wastewater discharging control. The developed method was then demonstrated in a water-stressed city (i.e., the City of Dalian), northeastern China. Three scenarios were proposed according to the city’s industrial plans. The results indicated that in the planning year of 2020 (a) the production of civilian-used steel ships and machine-made paper & paperboard would reduce significantly, (b) violation risk of chemical oxygen demand (COD) discharge under scenario 1 would be the most prominent, compared with those under scenarios 2 and 3, (c) the maximal total economic benefit under scenario 2 would be higher than the benefit under scenario 3, and (d) the production of rolling contact bearing, rail vehicles, and commercial vehicles would be promoted.

## Introduction

Water is one of the most vital natural resources in the world. In the past century, water in the earth was subject to significant changes due to rising water consumption that was driven by industrialization and urbanization^1–3^. Meanwhile, deterioration of water quality caused by industrial wastewater discharge has emerged in many developing countries^4^. For example, one-third of wastewater was discharged by industrial sectors in China according to environmental statistics of 2013^5^. Water managers of multiple jurisdictions are facing dual challenges from water resources shortage and water quality reduction. Such challenges will be aggravated in the future due to a significant increase in industrial water demand^6, 7^. Particularly, with the implementation of sustainable development plans for society, economy, and environment in China^8^, it is of importance to mitigate industrial impacts on water resources deficit and water quality decline in the near future^9–12^. Moreover, uncertain future conditions (e.g., unknown water inflows and diverse industrial activities) will continuously require novel methods to support decision making in industrial water resources management (IWRM) systems^13–15^.

Over the past decade, IWRM has attracted many studies^16^. The studies were mostly focused on water resources consumption of a single industrial sector or mill (e.g., a electroplating plant^17^, wine-producing industry^18^, iron and steel industry^19^, and process industries^20^). Similarly, industrial wastewater discharge was commonly focused within a specific industrial process or mill^21–23^. With the rapid development of urbanization and industrialization, conflicts among and within multiple water users (e.g., industrial and agricultural sectors) were considered^24^. Wang *et al*.^25^ evaluated the water reallocation alternatives from agriculture to industry based on multi-attribute decision support model. Also, many methods were adopted for supporting decision making in IWRM, such as mathematical programming^26^, multi-attribute decision support models^27^, and system analysis methods^18^. For example, Lin *et al*.^28^ evaluated water resource management strategies in high-tech industries of Taiwan by multiple-criteria decision analysis (MCDA). In terms of water allocation for multiple sectors, optimizing methods were adopted to identify desired strategies^29–31^. For example, Mushtaq and Moghaddasi^32^ proposed an optimization model to maximize economic benefits under a number of water-related, technical and administrative constraints. Li *et al*.^33^ proposed an interval multi-objective programming model to support the long-term industrial water resources management in Binhai New Area, Tianjin, China. Concurrently, it is economically and technically infeasible to maintain zero discharge of industrial wastewater. A lot of studies focused on treatment technologies for industrial wastewater (e.g., Arivoli *et al*.^34^) and waste load allocation for industrial wastewater discharge (e.g., Qin *et al*.^35^).

As responses to social, economic, and environmental disturbances, the following multi-level uncertainties posed a major challenge to decision making in the IWRM systems^36–38^: (a) variations in water demands and supply, as well as wastewater discharge may perturb signal identification and assessment^39^, (b) many forecasting data in social, economic, and environmental programs would not truly reflect real situations^40, 41^, and (c) the implications of randomness of data and vagueness of expert’s judgments may also lead to unexpected events^42–44^. Hence, water managers should consider uncertain features of the IWRM systems and recover equilibrium among industrial, agricultural, and residential water users^45–48^. Also, water pollutants discharged by industrial sectors may exceed the predetermined control targets, posing a potential violation risk for sustainable development^49^. To address the uncertainties and variations in the IWRM systems, a number of approaches (e.g., inexact optimization techniques, sensitivity analysis, statistical approaches and fuzzy sets theory) were developed^50–53^. Monte Carlo simulation (MCS) was widely used in data uncertainty analysis^54–57^. Fuzzy sets theory was applied for implicating vagueness of subjective judgments^58^. For example, Ren *et al*.^59^ developed a stochastic linear fractional programming model for desirable industrial structure under different risk probabilities of water resources.

Also, two-stage stochastic programming (TSP) was widely proved to be an effective tool for water allocation under uncertain conditions^50^. Meanwhile, an incident (e.g., failure to meet total allowable target on wastewater discharge) may be generated by multiple uncertainties of the IWRM systems. To quantitatively and systematically explore the likelihood of the incident, violation risk analysis should be incorporated^60, 61^. Previously, risk-based decision-making has been advocated for water resources management^62–64^. Studies on risk analysis of IWRM systems can be classified into the following two categories: (a) assessing health risk caused by industrial wastewater discharge^65–68^, and (b) analyzing water shortage risk arising from industrial water consumption^69, 70^. However, violation risk of wastewater discharge caused by various and uncertain industrial activities was rarely considered by previous studies.

To improve the capabilities of conventional methodologies for supporting industrial water resources management (IWRM) in uncertain and risky conditions, an integrated approach will be developed based on the combination of uncertainty analysis, violation analysis, and optimization model. The methodology could effectively reflect and address equilibrium between industrial water supply and demand in consideration of economic development and environmental protection. In detail, the paper will focus on the following aspects: (i) analyzing uncertainties of water demands, economic benefits, and wastewater discharge of future industrial activities, (ii) assessing violation risk of wastewater discharge by multiple industries, and (iii) allocating water resources to multiple industries under uncertain and risky conditions. The method can (a) systematically reflect and address complexities of IWRM systems, (b) effectively facilitate reflections of multiple uncertainties and risks of the system and incorporate them into a general optimization framework, and (c) successfully manage robust actions for industrial productions in consideration of water supply capacity and wastewater discharge. The developed method will then be demonstrated in a water-stressed city (i.e., city of Dalian) in northeastern China.

## Results and discussions

### COD discharge in industrial activities

Organic pollution by wastewater discharge from industrial activity affects humans and ecosystems worldwide^71^. In China, wastewater control is carried out based on the total allowable limits of water pollutants [e.g., chemical oxygen demand (COD)]. In detail, reduction of COD discharge of Dalian City would be no less than 2.04 Mt in 2015 compared with the discharge amount in 2010, according to the 12th Five-Year Plan for environmental protection.

**Figure 1:**
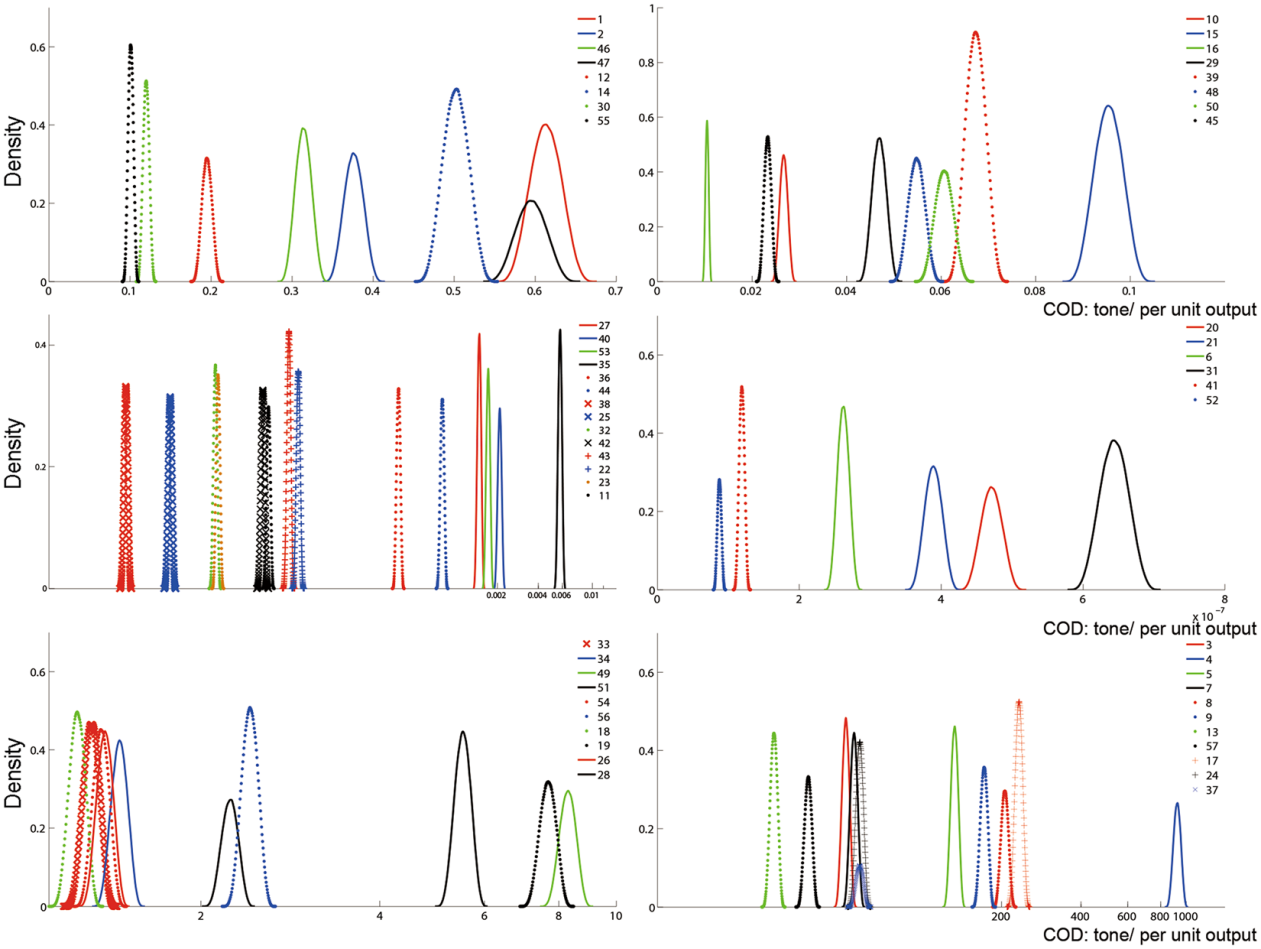
Uncertain feature of COD discharge in 57 industrial products of Dalian City. Based on the hybrid approach for data analysis, uncertain features of COD discharges in 57 industrial products of Dalian City can be described as probability density functions. Code numbers of products in the figure are listed in Table S3 of Supplementary Information.

The amounts of COD discharges for manufacturing industrial products in 2020 are estimated based on the national statistics^72^. Thus, the data quality scores of ${e}_{CO{D}_{j}}$ can be described as (4.8, 3.5, 4.2, 2.6, 3.1, 0.8) within level V. The uncertainty features of COD discharges in 57 industrial products are described in Fig. 1.

### Industrial water and wastewater management with discharge caps

There are multiple industrial plans in Dalian City (Table S5 of Supplementary Information). It is indicated that water resources could not fulfill all plans of industrial production. Thus, industrial plans have to be modified under the following three scenarios: (i) taking no account of the minimum growth rates for industries; (ii) considering development goals of top six industries in water-use efficiency perspective (i.e., garments, traditional Chinese medicines, rolling contact bearing, meta-cutting machines, rail vehicles, and civilian-used steel ships); and (iii) considering development goals of top six industries in economic benefit perspective (i.e., crude oil processed, rolling contact bearing, civilian-used steel ships, rail vehicles, light vehicles, and commercial vehicles). The final solutions contain the following three parts.

#### (1) Outputs of industrial products

**Figure 2:**
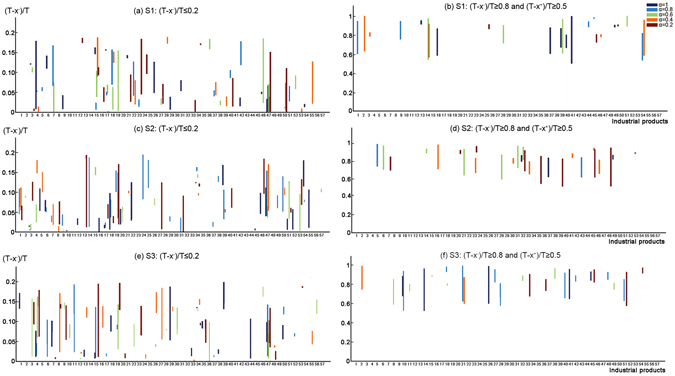
Output ratios of industrial products in 2020.

In this study, five *α*-cut levels are proposed for solving fuzzy parameter (i.e., discharge cap of COD in the future [i.e., ${\tilde{C}}_{COD}$]). The solutions for industrial production are shown in Table S13 of Supplementary Information. The outputs of civilian-used steel ships would decrease by more than 80% in at least three α-cut levels under the three scenarios. Meanwhile, the output of machine-made paper & paperboard would reduce more than 97% (Fig. 2). The output of metal shaping equipment would reduce less than 20% in at least one α-cut level. The details of the output strategies are described as the follows: a) under scenario 1, the outputs of seven types of industrial products (i.e., edible vegetable oil, fresh or chilled meat, compound floorboard, machine-made paper & paperboard, crude oil processed, civilian-used steel ships, and air conditioners) would reduce more than 80% in at least three α-cut levels; b) under scenario 2, the outputs of nine types of industrial products (i.e., salt, edible vegetable oil, machine-made paper & paperboard, crude oil processed, sodium carbonate, rolled steel, industrial boiler, civilian-used steel ships, and printers) would decrease by more than 80% in at least three α-cut levels; and c) under scenario 3, the outputs of three types of industrial products (i.e., fresh or chilled meat, rolled steel, and civilian-used steel ships) would decrease by more than 80% in at least three α-cut levels.

#### (2) Violation risks of wastewater discharge

**Figure 3:**
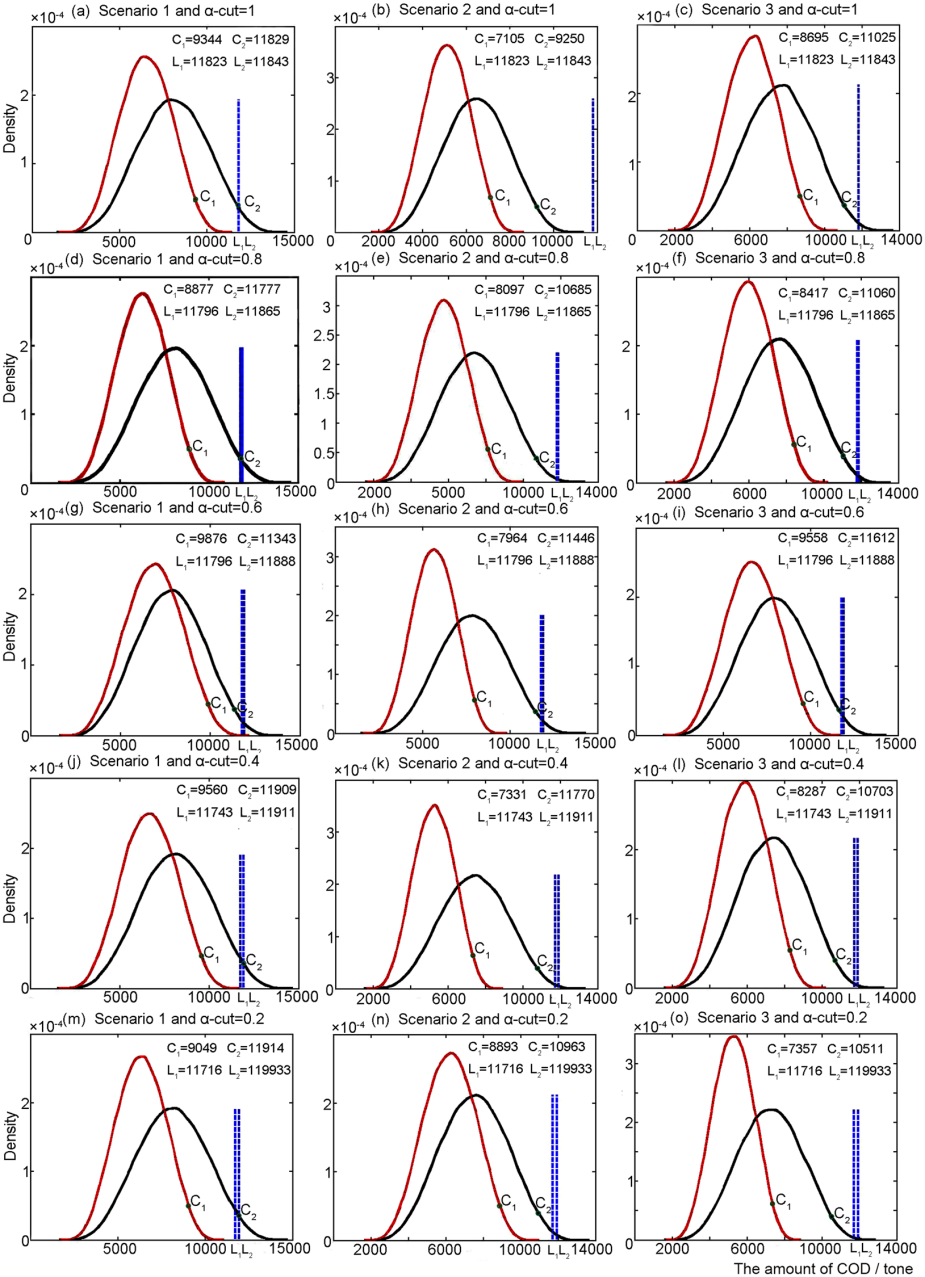
Violation risks of COD discharge in Scenarios 1 to 3. The parameters of C_1_ and C_2_ indicate probability of the incident (i.e., failure to meet total allowable target on wastewater discharge) would be 0.05. The parameters of L_1_ and L_2_ indicate the lower and upper bounds of discharge caps in COD.

Violation risk of COD discharge under scenario 1 would be more prominent than those under scenarios 2 and 3. Violation risk of COD discharge under scenario 3 would be more prominent than that under scenarios 2 in four α-cut levels. The results of violation risks are described as follows (Fig. 3): (a) the upper bounds of COD discharge for manufacturing industrial products would be the same as the maximum allowance limits under scenario 1; (b) the upper bounds of COD discharge for manufacturing industrial products would be less than the maximum allowance limits under scenario 2; and (c) the upper bounds of COD discharge for manufacturing industrial products would be less than the maximum allowance limits under scenario 3.

#### (3) Planning adjustment

**Figure 4:**
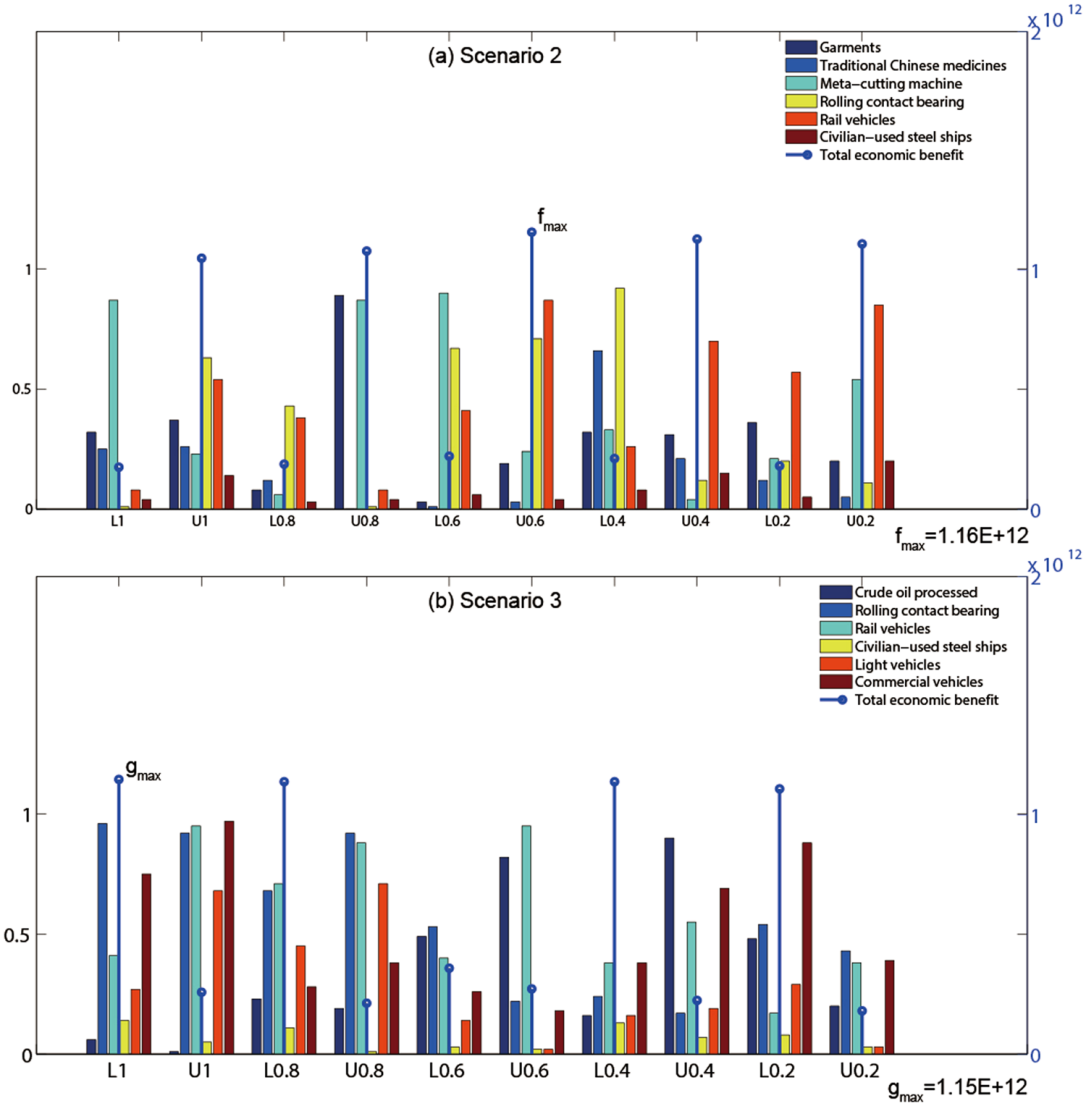
Planning modifications in scenarios 1 and 2. The letter of “L” represents the lower bound of interval numbers. The letter of “U” represents the upper bound of interval numbers. The numbers after the letters “L” and “U” are the multiple α-cut levels.

Under scenarios 2 and 3, multiple industrial products are chosen and modified (Fig. 4). Three types of industrial products (i.e., rolling contact bearing, rail vehicles, and civilian-used steel ships) are included in both scenarios. Generally, the total economic benefit under scenario 2 would be higher than that under scenario 3. Under scenario 2, manufacturing rolling contact bearing and rail vehicles would be promoted; conversely, manufacturing traditional Chinese medicines and civilian-used steel ships would not be encouraged. Under scenario 3, manufacturing rolling contact bearing and commercial vehicles would be promoted; conversely, manufacturing crude oil processed would not be encouraged.

## Conclusions

In this research, traditional methods for industrial water resources management (IWRM) were improved through the integration of operational research, uncertainty analysis, and violation risk analysis methods. This improved conventional industrial water resources management in (a) systematically reflecting multiple uncertain and risky features of IWRM systems, (b) adequately incorporating the features into industrial water and wastewater management, and (c) adequately managing robust actions for industrial productions in consideration of water supply capacity and wastewater discharge caps. This represented an improvement upon conventional methods for water resources management and violation risk analysis. The developed method was then demonstrated in Dalian. The following three scenarios were proposed according to different emphases in industrial development: (i) taking no account of the minimum growth rates for industries; (ii) considering development goals of top six industries in water-use efficiency perspective; and (iii) considering development goals of top six industries in economic benefit perspective. The results indicated that in the planning year (i.e., 2020) (a) the production of civilian-used steel ships and machine-made paper & paperboard would reduce significantly; (b) violation risk of COD discharge under scenario 1 would be the most prominent, compared with scenarios 2 and 3; and (c) the production of rolling contact bearing, rail vehicles, and commercial vehicles would be promoted.

## Methods

**Figure 5:**
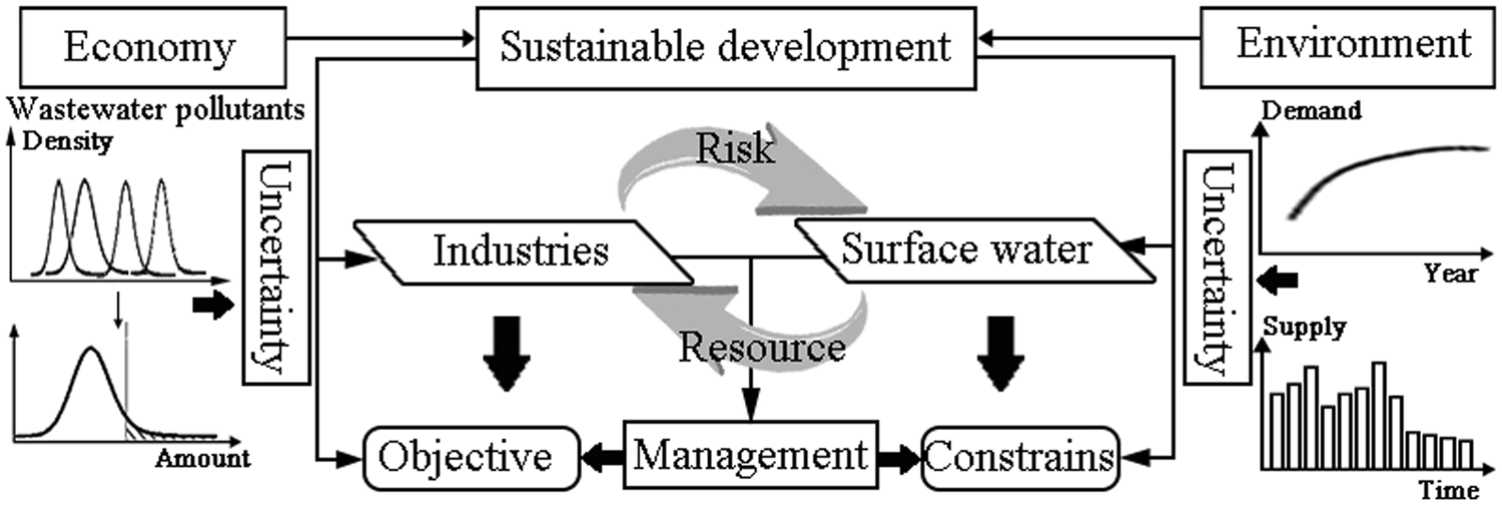
Industrial water and wastewater management under uncertain environmental-economic conditions.

It is a challenge for IWRM systems to realize conflicting goals on both economic benefits and wastewater discharges within multi-level uncertain conditions (Fig. 5)^73^. The multi-level complexities and uncertainties can be summarized as follows: (a) variations of water demands and supply, as well as wastewater discharge in the IWRM systems, (b) complexities of relationships between water demands and economic benefits of industries, (c) uncertainties of wastewater loadings among industries, and (d) likelihood that violation events occur.

**Figure 6:**
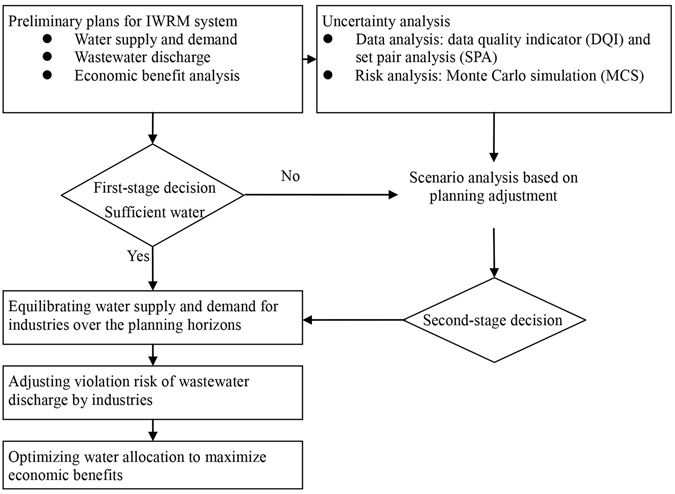
Industrial water resources management framework.

In this study, the following four parts were incorporated into IWRM (Fig. 6): (a) making preliminary plans for water allocations in the 1^st^-stage decision making, which contains water demand, wastewater discharge and economic benefit analyses, (b) analyzing data uncertainty in IWRM systems, and violation risks of wastewater discharge, (c) equilibrating water supply and demand, and adjusting the plans for the 2^nd^- stage decision making, and (d) formulating an optimization model (i.e., fuzzy inexact two-stage programming), with consideration of economic benefits, water demands, and pollutant reductions in the IWRM systems.

### Uncertainty analysis

Uncertainty analysis is composed by the following two methods: (a) a hybrid approach of data quality scores and fuzzy set pair analysis for analyzing parameter variations in the IWRM systems (e.g., water demands, wastewater discharge, and economic benefits); and (b) violation risk analysis based on Monte Carlo simulation for evaluating the likelihood of the incident (i.e., failure to meet wastewater discharge caps).

### Data analysis

**Figure 7:**
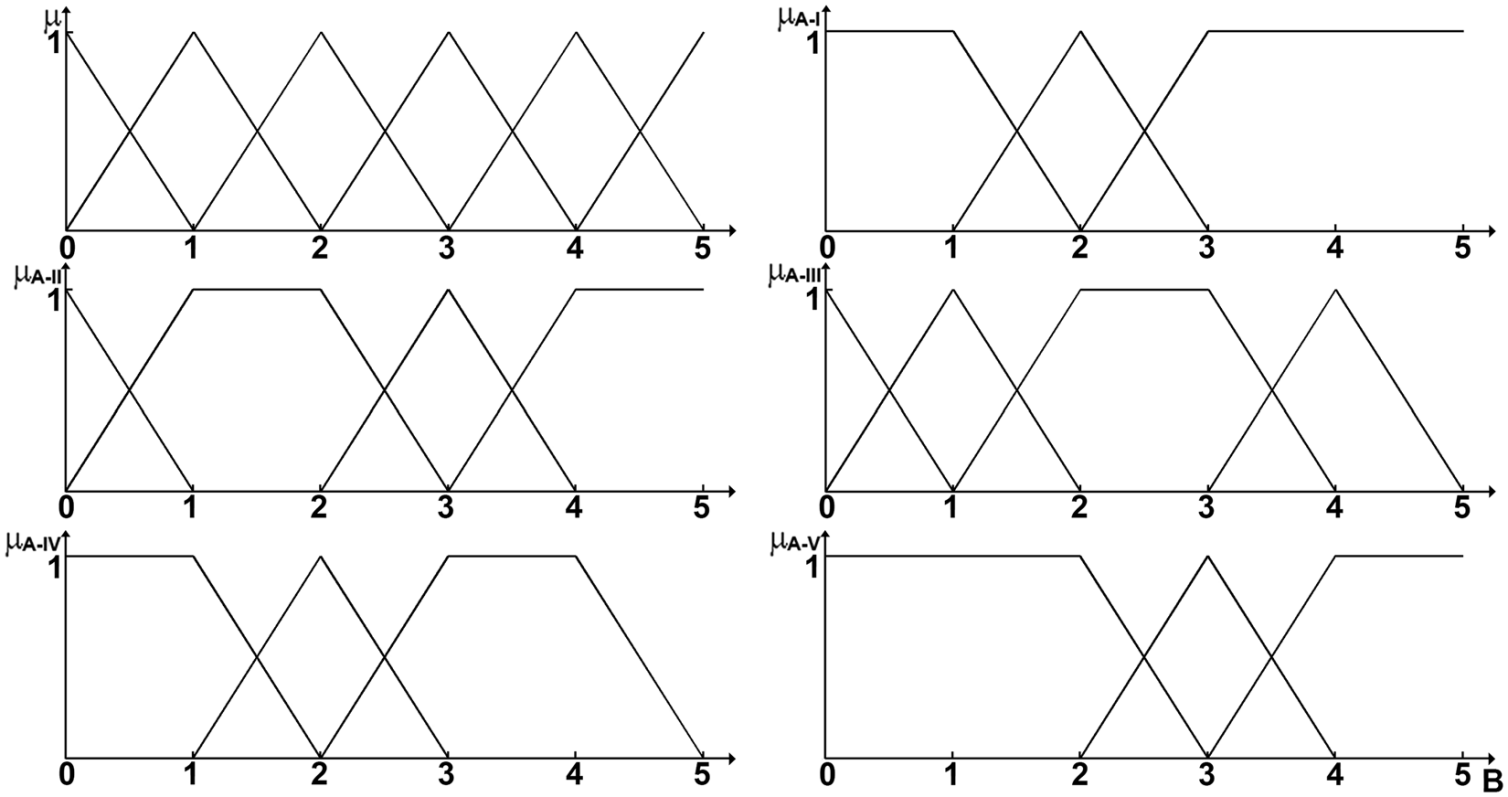
Levels of DQI (Set $\tilde{B}$).

In this research, approaches of data quality score and fuzzy sets pair analyses are adopted for facilitating uncertainty analysis. Based on the previous study by Yue *et al*.^74^, data quality scores are used to assess data quality from multiple perspectives^75^ (see Section S1.1 of Supplementary Information). Concurrently, a number of indicators will outweigh others in affecting data variations within meta-data vectors^76^. Thus, fuzzy set pair analysis (FSAP) is proposed to facilitate multi-criteria assessment of data quality^76^. The relationship between data quality scores and data quality levels can be defined as the matrix *H*_*A-B*_ in Equation 1:

1 $${H}_{A-B}=({H}_{{A}_{1}-B},{H}_{{A}_{2}-B},\ldots ,{H}_{{A}_{6}-B})=({b}_{1},{b}_{2},\ldots ,{b}_{6})$$

where data quality scores are described into set *A* [i.e., $A=({A}_{1},{A}_{2},\ldots ,{A}_{6})$], according to data quality pedigree matrix in Table S1 of Supplementary Information; levels of data quality are describe in set *B* [i.e., $B=(I,II,III,IV,V)$]; *H*_*A-B*_ is a set pair formed by sets *A* and *B*. In terms of the relationship between sets *A* and *B*, the connection degree of ${D}_{A-B}$ can be defined by the following equation^77, 78^:

2 $${D}_{A-{b}_{j}}=\frac{{S}_{k0}}{N}+\frac{{F}_{k1}}{N}{\tilde{I}}_{1}-\frac{{F}_{k2}}{N}{\tilde{I}}_{2}+\frac{{P}_{k3}}{N}J,\forall {b}_{j}$$

where ${D}_{A-{b}_{j}}$ is the connection degrees of data quality scores (i.e., set *A*) and a certain level of data quality (i.e., ${b}_{j}\in \{I,\mathrm{II},\mathrm{III},\mathrm{IV},V\}$); ${\tilde{I}}_{1}$ and ${\tilde{I}}_{2}$ are the uncertain coefficients between the ranges of  0 to 1, reflecting discrepancy degrees of set *A* and *B*; *J* is the contrary degree coefficient and specified as −*1*; *N* denotes the total number of element of matrix *H*_*A*−*B*_; ${S}_{k0}$ is the number of elements same with *b*_*j*_ in ${H}_{A-B}$; ${F}_{k1}$ is the number of elements same with *b*_*j*_ + *1* in ${H}_{A-B}$; ${F}_{k2}$ is the number of elements same with *b*_*j*_ + *2* in ${H}_{A-B}$; ${P}_{k3}$ is the number of elements that are more than *b*_*j*_ + *2* in ${H}_{A-B}$ (Fig. 7).

Equations 1 and 2 can make quantitative comparative analysis of attributes in identical, discrepancy and contrary aspects between data quality scores and data quality level^79, 80^. When ${\tilde{I}}_{1}$ and ${\tilde{I}}_{2}$ are assigned by the values between the range of 0 to1, they can reflect the proportion of certainty and uncertainty. The results of ${D}_{A-B}=({D}_{A-I},{D}_{A-II},{D}_{A-III},{D}_{A-IV},{D}_{A-V})$ can describe connection degree between data quality scores and levels. The bigger index in *D*_*A*−*B*_ (e.g., *D*_*A*−*III*_) means set *A* would be associated with the level (e.g., level III) in higher probability. Then, data uncertainty can be estimated by the following transformation matrix (Table 1).

**Table 1:** Transformation matrix.

set B	Beta distribution function	
	Shape parameters $(\alpha ,\beta )$	Range endpoints (±*r*%)
V	(5, 5)	10
IV	(3, 3)	20
III	(1, 1)	30
II	(1, 1)	40
I	(1, 1)	50

### Violation risk analysis

As wastewater discharge tend to be influenced by variation in densities and categories of industrial activities of the IWRM systems, the total amount of industrial wastewater may exceed the maximum allowable limit (i.e., violation of wastewater discharge). Depending on what is known and not known, violation risk of wastewater discharge can be analyzed based on uncertainty bounds which exceed classical deterministic margins^80–83^. To accommodate this kind of uncertainty, there are two main techniques, i.e., probability and possibility theories for cognitive uncertainties^44^. In this paper, based on the data analysis, the amount of COD discharge by a certain industry in future year could be estimated by random variables with beta distribution function, i.e., ${e}_{CO{D}_{j}}$. Thus, total amount of COD discharge can be described by Equation 3:

3 $${E}_{COD}^{\pm }=({E}_{COD}^{-},{E}_{COD}^{+})=(\sum _{j}{e}_{CO{D}_{j}}\,{y}_{j}^{-},\sum _{j}{e}_{CO{D}_{j}}\,{y}_{j}^{+})$$

where ${E}_{COD}^{\pm }$ is total amount of COD discharge; ${e}_{CO{D}_{j}}$ is amount of COD discharge by the *j* ^*th*^ industry per unit production; ${y}_{j}^{\pm }$ is the output of the *j* ^*th*^ industry. The violation risk can be described by the relationship between discharge amount and control target, [i.e., $P({E}_{COD}\ge {\tilde{C}}_{COD})$] (Equations 4 and 5)^84^:

4 $${R}_{COD}=P({E}_{COD}^{\pm }\ge {\mathop{C}\limits^{ \sim }}_{COD})=Max\{{\int }_{{E}_{COD}^{-}\ge {\mathop{C}\limits^{ \sim }}_{COD}}G{(X)}^{-}dX,{\int }_{{E}_{COD}^{+}\ge {\mathop{C}\limits^{ \sim }}_{COD}}G{(X)}^{+}dX\}={\int }_{{E}_{COD}^{+}\ge {\mathop{C}\limits^{ \sim }}_{COD}}G{(X)}^{+}dX$$

5 $$G{(X;\alpha ,\beta ,a,b)}^{+}=[\frac{1}{b-a}]\times [\frac{{\rm{\Gamma }}(\alpha +\beta )}{{\rm{\Gamma }}(\alpha )+{\rm{\Gamma }}(\beta )}]\times {[\frac{X-a}{b-a}]}^{\alpha -1}\times {[\frac{b-X}{b-a}]}^{\beta -1}$$

where $G{(X)}^{-}$ and $G{(X)}^{+}$ are the probability density functions of ${E}_{COD}^{-}$ and ${E}_{COD}^{+}$; *α* and *β* are distribution shape parameters; *a* and *b* are range endpoints based on the results in data analysis. Meanwhile, the probability density functions of $G{(X)}^{-}$ and $G{(X)}^{+}$ are applied by Monte Carlo simulation upon 50,000 iterations to analyze data ranges^85^.

### Industrial water allocation with wastewater caps

Consider a problem in which a water manager is in charge of supplying water resources to industries for producing multiple products in a city (e.g., cars, garments, and furniture)^86, 87^. The industries want to expand their activities and need to know how much water they can obtain. The water manager can formulate the problem through maximizing the economic benefits of industrial products with consideration of economic benefits, water demands, and wastewater discharge in the IWRM systems. An inexact risk management optimization model is effective for water allocation in multiple users over time and uncertain parameters^88^. Thus, the problem can be described by the following equations:

6 $${\rm{\max }}\,{f}^{\pm }=\sum _{j=1}^{n}{a}_{j}^{\pm }{T}_{j}-\sum _{j=1}^{n}{d}_{j}^{\pm }{x}_{j}^{\pm }$$

s.t.

7 $${T}_{j}\ge {x}_{j}^{\pm }\ge 0,\forall j$$

8 $$\sum _{l}{C}_{l}\ge \sum _{j=1}^{n}{t}_{j}^{\pm }({T}_{j}-{x}_{j}^{\pm })+{W}_{A}^{\pm }+{W}_{R}^{\pm }$$

9 $$\sum _{l}{q}_{l}\ge \sum _{j=1}^{n}{t}_{j}^{\pm }({T}_{j}-{x}_{j}^{\pm })+{W}_{A}^{\pm }+{W}_{R}^{\pm }$$

10 $${T}_{j^{\prime} }-{x}_{j^{\prime} }^{\pm }\ge (1+{\varepsilon }_{j^{\prime} }^{\pm }){Y}_{0},\forall j^{\prime} =1,2,\cdots ,k$$

11 $$\sum _{j=1}^{n}{a}_{j}^{\pm }({T}_{j}-{x}_{j}^{\pm })\ge {(1+{\eta }^{\pm })}^{N}\sum _{j=1}^{n}{a}_{j0}{T}_{j0}$$

12 $${R}_{COD}=P({E}_{COD}^{\pm }\ge {\tilde{C}}_{COD})\le 5 \% $$

where *f*: Economic benefit of industrial production;

${a}_{j}^{\pm }$: Profit of per unit output for the ${j}^{th}$ industrial product;

${T}_{j}$: Preliminary plan of the ${j}^{th}$ industry in the first-stage decision making;

${d}_{j}^{\pm }$: Economic loss of the ${j}^{th}$ industry when 1 m^3^ water would not be delivered;${x}_{j}^{\pm }$: Shortage of output on the ${j}^{th}$ industrial product in the second-stage decision making;

${C}_{l}$: Water supply capacity of the *l* ^*th*^ water source;

${t}_{j}^{\pm }$: Water-use quota for the ${j}^{th}$ industrial product;

${W}_{A}^{\pm }$: Amount of water for agricultural crop;

${W}_{R}^{\pm }$: Amount of water for social user (e.g., residents, schools, and hospitals);

*q*_*l*_: total amount of seasonal flow (m^3^) of the *l* ^*th*^ river;

${\varepsilon }_{j^{\prime} }^{\pm }$: Minimum growth rate for the ${j^{\prime} }^{th}$ product in the industrial plan;

${Y}_{0}$: The output of the ${j^{\prime} }^{th}$ product in the base year;

${\eta }^{\pm }$: Annual economic growth rate of the city;

*N*: Number of years between the planning year and base year;

${T}_{j0}$: Output of the ${j}^{th}$ industrial product in the base year;

${a}_{j0}$: Profit of per unit output for the ${j}^{th}$ industrial product in the base year.

## Electronic supplementary material

Supplementary Information
