# Supplementary material for: Industrial water resources management based on violation risk analysis of the total allowable target on wastewater discharge
Source: Sci Rep. 2017 Jul 11;7:5055. doi: 10.1038/s41598-017-04508-9 (PMC5506039; doi:10.1038/s41598-017-04508-9)
Supplement: Supplementary file 1 — Supplementary Information [file 41598_2017_4508_MOESM1_ESM.doc]

**Supplementary Information:**

**Industrial water resources management based on violation risk analysis of the total allowable target on wastewater discharge**

Wencong Yue, Yanpeng Cai^*^, Linyu Xu, Zhifeng Yang^*^, Xin’An Yin, Meirong Su

**S1 Methods**

**S1.1 Data quality score analysis**

Traditional data quality score are designed in terms of several aspects (i.e., supplier independence, acquisition method, data representativeness, age, geographical correlation, and technological correlation) to provide scores (i.e., 1 to 5). Detail of the method should be referred to May and Brennan^1^, and Lewandowska et al.^2^. In this research, data quality scores are evaluated [e.g., (*A_1_*, *A_2_*, *A_3_*, *A_4_*, *A_5_*, *A_6_*) = (2.2, 1.6, 4.2, 3.5, 2.3)] according to the six aspects (Table S1).

**Table S1.** Data quality pedigree matrix.

| Quality level (set B) | Quality  Score | Data quality indicators | | | | | |
| --- | --- | --- | --- | --- | --- | --- | --- |
|  |  | Supplier independence | Acquisition  method | Data representativeness | Age | Geographical  correlation | Technological  correlation |
|  |  | A_1_ | A_2_ | A_3_ | A_4_ | A_5_ | A_6_ |
| V | [4, 5] | Verified data from independent source | Directly measured data | Representative data from a sufficient sample of sites over an adequate period to even out normal fluctuations | (0, 3] | from the exact area | From process studied of the exact company with the exact technology |
| IV | [3, 4) | Verified data from enterprise with interest in the study | Calculated data based on measurements | Representative data from a smaller number of sites but for an adequate period | (3, 6] | Average data | From process studied of company with similar technology |
| III | [2, 3) | Independent source but based on unverified information | Calculated data partly based on assumptions | Representative data  from an adequate number of sites but for a shorter period | (6, 10] | From an area with similar production conditions | From process studied of company with different technology |
| II | [1, 2) | Unverified information from irrelevant enterprise | Qualified estimation by experts | Data from a smaller number of sites for a shorter period, or incomplete data from an adequate number of sites and periods | (10, 15] | From an area with slightly similar production conditions | From process related of company with similar technology |
| I | (0, 1) | Unverified information from enterprise interested in the study | Non-qualified estimation | Representativeness unknown or incomplete data from insufficient sample of sites and/or for a shorter period | ≥15 | Unknown area | From process related of company with different technology |

Source: Wang and Shen^3^

# S1.2 Solution methods

# (1) Violation risk analysis

Monte Carlo sampling (MCS) can estimate the violation risks by simulating the random variables. Assuming $\tilde{H}_{COD}^{\pm}=E_{COD}^{\pm}-\tilde{C}_{COD}$, the violation risks under certain *α*- cut can be described as follows^4^:

$R_{COD-\alpha}^{\pm}=\left[\frac{1}{N}\sum_{j=1}^{N} I\left(H_{COD-\alpha L}^{-}\right)\text{， }\frac{1}{N}\sum_{j=1}^{N} I\left(H_{COD-\alpha R}^{+}\right)\right]$ (S1)

where *N* is the total number of simulations conducted and *I(H)* is an indicator for failure as Equation S2:

$I\left(H\right)=\left\{\begin{matrix} 1,\text{ }if\text{ }H\geq0 \\ 0,\text{ }if\text{ }H<0 \end{matrix}\right.$ (S2)

Latin hypercube sampling (LHS) is a stratified random procedure that can efficiently sample variables from their multivariate distributions^5^. The LHS strategy in this research is to insert the sampling points of wastewater discharges to examining constraints of programming models^6^. The algorithm includes the following four steps: (i) producing sampling matrixes of industrial densities by LHS, (ii) examining the violation risk and other constraints of the optimization model in the matrix condition, (iii) checking whether matrix fulfill the maximizing objective, and (iv) obtaining optimal solutions for IWRM.

# (2) Interval programming model

Let us consider the following linear programming problem with interval parameters:

$\text{Max }z^{\pm}\text{ }=\text{ }c^{\pm}x^{\pm}$ (S3)

s.t.

$a_{i}^{\pm}x^{\pm}\text{ }\leq\text{ }b_{i}^{\pm},\text{ }i\text{ }=\text{ }1,\text{ }2,\text{ }\cdots,\text{ }m$ (S4)

$z^{\pm}\text{ }>\text{ }0$ (S5)

$x^{\pm}\text{ }\geq\text{ }0$ (S6)

According to the algorithms for objective to maximize $z^{\pm}$^7,8^, the solution for interval linear programming (ILP) model can be obtained through a two-step method, where a sub-model corresponding to $z^{-}$ is first formulated and solved, and then the relevant sub-model corresponding to $z^{+}$ can be formulated based on the solution of the first sub-model. In detail, the first sub-model can be formulated as follows.

$\text{Max }z^{+}=\sum_{j=1}^{k_{1}} c_{j}^{+}x_{j}^{+}+\sum_{j=k_{1}+1}^{n} c_{j}^{+}x_{j}^{-}$ (S7)

s.t.

$\sum_{j=1}^{k_{1}} \left|a_{ij}\right|^{-}Sign(a_{ij}^{-})x_{j}^{+}+\sum_{j=k_{1}+1}^{n} \left|a_{ij}\right|^{+}Sign(a_{ij}^{+})x_{j}^{-}\leq b_{j}^{+},\text{ }\forall j$ (S8)

$x_{j}^{+}\text{, }x_{j}^{-}\geq0$ (S9)

where $x_{j}^{\pm}$, $j\text{ }=\text{ }1,\text{ }2,\text{ }\cdots,\text{ }k_{1}$ are interval variables with positive coefficients in the objective function and $x_{j}^{\pm}$, $j\text{ }=\text{ }k_{1}+1,\text{ }k_{1}+2,\text{ }\cdots,\text{ }n$ are interval variables with negative coefficients in the objective function. Thus, solutions of $x_{j\text{ }opt}^{+}$ ($j=1,\text{ }2,\text{ }\cdots,\text{ }k_{1}$) and $x_{j\text{ }opt}^{-}$ ($j\text{ }=\text{ }k_{1}+1,\text{ }k_{1}+2,\text{ }\cdots,\text{ }n$) can be obtained through solving sub-model 4. Then the sub-model corresponding to $f^{-}$ can be formulated as follows:

$\text{Max }z^{-}=\sum_{j=1}^{k_{1}} c_{j}^{-}x_{j}^{-}+\sum_{j=k_{1}+1}^{n} c_{j}^{-}x_{j}^{+}$ (S10)

s.t.

$\sum_{j=1}^{k_{1}} \left|a_{ij}\right|^{+}Sign(a_{ij}^{+})x_{j}^{-}+\sum_{j=k_{1}+1}^{n} \left|a_{ij}\right|^{-}Sign(a_{ij}^{-})x_{j}^{+}\leq b_{j}^{-},\text{ }\forall j$ (S11)

$x^{\pm}\text{ }\geq\text{ }0$ (S12)

$0\leq x_{j}^{-}\leq x_{j\text{ }opt}^{+},\text{ }\forall j=1,\text{ }2,\text{ }\cdots,\text{ }k_{1}$ (S13)

$x_{j}^{+}\geq x_{j\text{ }opt}^{-},\text{ }\forall j=k{}_{1}+1,\text{ }k_{2}+2,\text{ }\cdots,\text{ }n$ (S14)

Thus, the risk management of industrial water-resource can be formulated as the following equations.

$\max f^{+}=\sum_{j=1}^{n} a_{j}^{+}T_{j}-\sum_{j=1}^{n} d_{j}^{-}x_{j}^{-}$ (S15)

s.t.

$T_{j}\geq x_{j}^{-}\geq0,\text{ }\forall j$ (S16)

$\sum_{l} C_{l}\text{ }\geq\text{ }\sum_{j=1}^{n} t_{j}^{-}\left(T_{j}-x_{j}^{-}\right)+W_{A}^{-}+W_{R}^{-}$ (S17)

$\sum_{l} q_{l}\text{ }\geq\text{ }\sum_{j=1}^{n} t_{j}^{-}\left(T_{j}-x_{j}^{-}\right)+W_{A}^{-}+W_{R}^{-}$ (S18)

$T_{j'}-x_{j'}^{-}\geq\text{ (1+}\varepsilon_{j'}^{+}\text{)}Y_{j'0}\text{， }\forall j'=1,\text{ }2,\text{ }\cdots,\text{ }k$ (S19)

$\sum_{j=1}^{n_{1}} a_{j}^{+}\left(T_{j}-x_{j}^{-}\right)\geq\text{(1+}\eta^{+}\text{)}^{N}\sum_{j=1}^{n_{1}} a_{j0}T_{j0}$ (S20)

$R_{COD}\leq5\text{\%}$ (S21)

$\max f^{-}=\sum_{j=1}^{n} a_{j}^{-}T_{j}-\sum_{j=1}^{n} d_{j}^{+}x_{j}^{+}$ (S22)

s.t.

$T_{j}\geq x_{j}^{+}\geq0,\text{ }\forall j$ (S23)

$\sum_{l} C_{l}\text{ }\geq\text{ }\sum_{j=1}^{n} t_{j}^{+}\left(T_{j}-x_{j}^{+}\right)+W_{A}^{+}+W_{R}^{+}$ (S24)

$\sum_{l} q_{l}\text{ }\geq\text{ }\sum_{j=1}^{n} t_{j}^{+}\left(T_{j}-x_{j}^{+}\right)+W_{A}^{+}+W_{R}^{+}$ (S25)

$T_{j'}-x_{j'}^{+}\geq\text{ (1+}\varepsilon_{j'}^{-}\text{)}Y_{j''0}\text{， }\forall j'=1,\text{ }2,\text{ }\cdots,\text{ }k$ (S26)

$\sum_{j=1}^{n_{1}} a_{j}^{-}P_{j}^{-}\left(T_{j}-x_{j}^{+}\right)\geq\text{(1+}\eta^{-}\text{)}^{N}\sum_{j=1}^{n_{1}} a_{j0}T_{j0}$ (S27)

$R_{COD}\leq5\text{\%}$ (S28)

$x_{j}^{+}\geq x_{j\text{ }opt}^{-},\text{ }\forall j$ (S29)

**S2 Applications**

As a city in north-eastern China, Dalian City is located at the southern tip of the Liaodong Peninsula, with the Yellow Sea to the east and the Bo Sea to the west. The city is composed of eight districts, i.e., Municipal zone, Jinzhou, Pulandian, Wafangdian, Changxingdao, Zhuanghe, Huayuankou, and Changhai. Water supply mostly depends on surface water transferred by rivers, reservoirs, and links, such as Biliu River, Yingna River, and Dasha River. The annual average precipitation in the city is from 600 to 800 mm. In past years, water resources were mainly supplied by local rivers in the city (e.g., Yingna, Biliu, and Dasha Rivers). However, Dalian has an inherently small fresh water supply. The per capita water resources supply in Dalian City was less than a quarter of national average level^9^. With the development of the population and economy, local water sources would not fulfil the demands of the city. According to the Dalian water sources plan, nearly 50% of water resource would be supplied by water source outside of the city in 2030 (i.e., Hun River in Fushun City)^10^.

**S2.1 Industrial water and wastewater management with discharge caps**

The inexact risk management optimization model for industrial water and wastewater management of Dalian City is presented as follows:

$\max f^{\pm}=\sum_{j=1}^{57} a_{j}^{\pm}T_{j}-\sum_{j=1}^{57} d_{j}^{\pm}x_{j}^{\pm}$ (S30)

s.t.

$T_{j}\geq x_{j}^{\pm}\geq0,\text{ }\forall j$ (S31)

$\sum_{l=1}^{7} C_{l}\geq\sum_{j=1}^{57} t_{j}^{\pm}\left(T_{j}-x_{j}^{\pm}\right)+W_{A}^{\pm}+W_{R}^{\pm}$ (S32)

$\sum_{l=1}^{7} q_{l}\geq\sum_{j=1}^{57} t_{j}^{\pm}\left(T_{j}-x_{j}^{\pm}\right)+W_{A}^{\pm}+W_{R}^{\pm}$ (S33)

$T_{j'}-x_{j'}^{\pm}\geq\text{ (1+}\varepsilon_{j'}^{\pm}\text{)}Y_{j'0}\text{， }\forall j'=1,\text{ }2,\text{ }\cdots,\text{ 22}$ (S34)

$\sum_{j=1}^{57} a_{j}^{\pm}P_{j}^{\pm}\left(T_{j}-x_{j}^{\pm}\right)\geq\text{(1+}\eta^{\pm}\text{)}^{10}\sum_{j=1}^{57} a_{j0}T_{j0}$ (S35)

$R_{COD}=P\left(E_{COD}^{\pm}\geq\tilde{C}_{COD}\right)\leq5\%$ (S36)

**S2.2 Data preparation in Case study**

**S2.2.1 Water supply**

Water sources in Dalian City are composed by surface, ground and recycled water. It is estimated that the amounts of ground, desalinated and recycled water would be 57.5, 310.8, and 387.5 Mt in 2020. The amount of surface water available for the city is described in Table S2. According to the regional development plans for Dalian water resources management^11^, Hun River in Fushun City will become a main water source outside of Dalian city. It is estimated that 0.45 billion m^3^ surface water will be supplied to Dalian City in 2020.

**Table S2.** Water availability and supply capacity of surface water in Dalian City.

Unit: 10^8^ m^3^

| Rivers | Amount of runoff | Water supply capacity |
| --- | --- | --- |
|
| Biliu River (upper reach of Biliuhe reservoir) | 6.0 | 4.5 |
| Yingna River (upper reach of Yingnahe reservoir) | 3.1 | 2.4 |
| Fuzhou River (upper reach of Dongfeng reservoir) | 0.7 | 0.7 |
| Fuzhou River (upper reach of Songshu reservoir) | 1.3 | 0.5 |
| Dasha River (upper reach of Liuda reservoir) | 2.0 | 0.5 |
| Zhuang Rriver (upper reach of Zhuwei reservoir) | 2.6 | 0.8 |
| Total | 15.6 | 9.4 |

**S2.2.2 Water consumption**

Fifty seven categories of industrial products are manufactured in Dalian City (Table S3). Water consumption for industrial products are estimated by water consumption quota for industries (Table S4) based on the corresponding national regulations and rules. According to the industrial planning of Dalian, in the next five years (from 2015 to 2020), the city will focus on the development of manufacture of machinery, petrochemical industry, harbor logistics industry. The plans for main industrial products of Dalian are shown in Table S5.

**Table S3.** Code numbers of products in case study.

| Number | Type of product | Number | Type of product | Number | Type of product |
| --- | --- | --- | --- | --- | --- |
| 1 | Salt | 20 | Aquafortis | 39 | Industrial boiler |
| 2 | Electricity | 21 | Synthetic ammonia | 40 | Internal combustion engines |
| 3 | Edible vegetable oil | 22 | Chemical pesticide | 41 | Meta-cutting machines |
| 4 | Fresh or chilled meat | 23 | Paint | 42 | Cranes |
| 5 | Dairy products | 24 | Plastic in primary forms | 43 | Rolling contact bearing |
| 6 | Beer | 25 | Synthetic detergents | 44 | Metal smelting equipments |
| 7 | Soft drinking | 26 | Traditional Chinese medicines | 45 | Metal shaping equipment |
| 8 | Chemical fiber | 27 | Tires | 46 | Rail vehicles |
| 9 | Yarn | 28 | Plastic products | 47 | Civilian-used steel ships |
| 10 | Cloth | 29 | Cement clinker | 48 | Household refrigerators |
| 11 | Garments | 30 | Cement | 49 | Commercial refrigerators |
| 12 | Leather shoes or boots | 31 | Commercial concrete | 50 | Air conditioners |
| 13 | Leather Garment | 32 | Plate glass | 51 | Printers |
| 14 | Wood floorboard | 33 | Crude steel | 52 | Semiconductor integrated circuits |
| 15 | Compound floorboard | 34 | Rolled steel | 53 | Integrated circuit chip |
| 16 | Furniture | 35 | Copper | 54 | Color television sets |
| 17 | Machine-made paper & paperboard | 36 | Aluminum | 55 | Video disc player |
| 18 | Crude oil processed | 37 | Metal container | 56 | Light vehicles |
| 19 | Sodium carbonate | 38 | Metal cutting tools | 57 | Commercial vehicles |

**Table S4.** Water quotas for industries according to the national standards.

| Industrial products | Water consumption quotas | | | Industrial Products | Water consumption quotas | | |
| --- | --- | --- | --- | --- | --- | --- | --- |
|  | - | + | Unit |  | - | + | Unit |
| 1 | 1 | | m^3^/t | 30 | 0.15 | | m^3^/t |
| 2 | 2.4 | 3.2 | m^3^/MWh | 31 | 1 | 2 | m^3^/m^3^ |
| 3 | 0.4 | 1.2 | m^3^/t | 32 | 0.4 | 0.6 | m^3^/t |
| 4 | 5 | 5.5 |  | 33 | 4.5 | 7 |  |
| 5 | 5 | 16 |  | 34^#^ | 2 | 4 |  |
| 6 | 3.09 | 3.78 | m^3^/1000L | 35 | 6.58 | |  |
| 7 | 2 | 2.8 | m^3^/t | 36^§^ | 9 | |  |
| 8 | 19 | |  | 37^§^ | 13 | |  |
| 9 | 20 | |  | 38 | 13 | | m^3^/100 units |
| 10 | 1.3 | | m^3^/100m | 39 | 60 | | m^3^/t |
| 11 | 90 | | m^3^/10000 units | 40 | 980 | 1300 | m^3^/10000KW |
| 12 | 47 | |  | 41 | 125 | | m^3^/unit |
| 13^#^ | 2250 | |  | 42 | 13 | | m^3^/t |
| 14^§^ | 6 | | m^3^/km^2^ | 43 | 35 | | m^3^/10000 units |
| 15^§^ | 20 | |  | 44^&^ | 24 | | m^3^/t |
| 16 | 0.2 | 0.5 | m^3^/unit | 45 | 24 | |  |
| 17 | 20 | 30 | m^3^/t | 46 | 3500 | | m^3^/unit |
| 18 | 0.58 | |  | 47^‡^ | 70 | 110 | m^3^/ tonnage |
| 19 | 15 | |  | 48 | 0.5 | | m^3^/unit |
| 20 | 3.3 | 4.5 |  | 49 | 0.5 | |  |
| 21 | 13 | 27 |  | 50 | 46 | |  |
| 22 | 50 | 98 |  | 51^§^ | 11 | | m^3^/10000 units |
| 23 | 2 | 10 |  | 52 | 0.2 | 2.4 |  |
| 24 | 14.5 | 16.5 |  | 53 | 1.4 | 1.7 | m^3^/unit |
| 25 | 3 |  |  | 54 | 0.05 | |  |
| 26 | 200 |  |  | 55^§^ | 180 | | m^3^/10000 units |
| 27 | 170 |  |  | 56 | 4.5 | | m^3^/unit |
| 28 | 3 | |  | 57 | 4.5 | |  |
| 29^†^ | 0.2 |  |  |  |  |  |  |

Source: ^#^AQTSGD^12^; ^§^WASZ^13^; ^†^WRDXJ^14^; ^‡^AQTSFJ^15^; ^&^AQTSYN^16^; other industrial products are referred to AQTSLN^17^.

**Table S5.** Industrial plans of Dalian City.

| Industrial Products | Base year (2010) | Planning year (2020) | Unit |
| --- | --- | --- | --- |
| Chemical fiber | 2.4 | 9.21 | 10000 t* |
| Yarn | 2.3 | 8.82 | 10000 t |
| Cloth | 752 | 2884.57 | 10000 m |
| Garments | 12351.7 | 47379.41 | 10000 units |
| Crude oil processed | 2652.9 | 4000 | 10000 t |
| Traditional Chinese medicines | 164 | 629.08 | t |
| Rolled steel | 183.8 | 705.03 | 10000 t |
| Meta-cutting machine | 52024 | 188390.37 | Unit |
| Rolling contact bearing | 14750.9 | 43267.29 | 10000 units |
| Rail vehicles | 589 | 2157.51 | Unit |
| Civilian-used steel ships | 636.1 | 2330.04 | 10000 tonnage |
| Household refrigerators | 44.6 | 127.76 | 10000units |
| Commercial refrigerators | 24.4 | 69.9 |
| Air conditioners | 163.8 | 469.23 |
| Printer | 85.1 | 243.78 |
| Color television sets | 247 | 707.58 |
| Video disc player | 298.7 | 855.68 |
| Light vehicles | - | 0.9 |
| Commercial vehicles | - | 0.45 |

*Note: 1 t = 1 tonne = 1000 kg; 1Mt=1000, 000 t.

Concurrently, water consumption quotas for urban and rural residents are 85 to 135 and 65 to 85 L per person in one day^17^. Water demands of residents in base and planning years are described in Table S6. Meanwhile, water demands of secondary and tertiary industries in Dalian City are listed in Table S7.

**Table S6.** The amount of water consumption for urban and rural residents.

Unit: Population × 10^4^ people; Consumption 10^4^ m^3^

|  | 2010 | | | | 2020 | | | | | |
| --- | --- | --- | --- | --- | --- | --- | --- | --- | --- | --- |
|  | Population | | Consumption | | Population | | Consumption | | | |
|  | Urban | Rural | Urban | Rural | Urban | Rural | Urban | | Rural | |
|  |  |  |  |  |  |  | Lower | Upper | Lower | Upper |
| Municipal zone | 253.6 | 10.0 | 9948 | 306 | 320.2 | 0 | 9349 | 15777 | 0 | 0 |
| Jinzhou | 56.8 | 23.7 | 2534 | 751 | 164.0 | 0 | 4790 | 8083 | 0 | 0 |
| Pulandian | 29.8 | 68.9 | 1081 | 1197 | 83.2 | 34.1 | 2428 | 4098 | 809 | 1059 |
| Wafangdian | 54.2 | 51.5 | 1715 | 1160 | 82 | 37.4 | 2394 | 4040 | 888 | 1161 |
| Changxingdao | 15.0 | 5.0 | 571 | 136 | 45 | 1 | 1314 | 2217 | 24 | 31 |
| Zhuanghe | 31.1 | 55.7 | 1158 | 1251 | 61.3 | 31.2 | 1790 | 3021 | 740 | 968 |
| Huayuankou | 1.6 | 4.8 | 59 | 145 | 25.7 | 1.8 | 750 | 1266 | 43 | 56 |
| Changhai | 3.9 | 3.4 | 110 | 80 | 11.6 | 1.6 | 338 | 570 | 37 | 48 |
| Total | 446 | 223 | 17176 | 5026 | 793 | 107 | 23153 | 39072 | 2541 | 3323 |

**Table S7.** Water demands of secondary and tertiary industries in Dalian City.

Unit: 10^4^m^3^

| Other industries | | 2020 |
| --- | --- | --- |
| Secondary industry | Agricultural | 50648 |
|  | Animal husbandry and fishery | 4481 |
|  | Construction | 3782 |
| Tertiary industry | | 25437 |

**S2.2.3 Wastewater discharge**

The amount of wastewater discharges from industries in Dalian City was estimated based on the average amount of wastewater discharges in China (Equation S37). The results of them are listed in Table S8.

$e_{COD\_j}=\frac{EP_{j}\times t_{j0}}{\sum_{j=1}^{n'} \left(EP_{j}\times t_{j0}\right)}\times e'_{COD\_i}$ (S37)

Where *EP_j_* is the export price of *j^th^* product, *t_j0_* is the output of *j^th^* industrial product in base year, $n'$ is the number of products belonging to the industry, $e'_{COD\_i}$is the amount of COD discharge of *i^th^* industry in China.The patterns of COD discharge in future are estimated by national industrial wastewater discharge according to NBS and MEP (2013).

**Table S8.** The amount of COD discharged by the industries of Dalian City in 2012.

Unit: t/ per unit output

| Industrial Products | *COD* | Industrial Products | *COD* | Industrial Products | *COD* |
| --- | --- | --- | --- | --- | --- |
| 1 | 0.61 | 20 | 4.71×10^-7^ | 39 | 0.07 |
| 2 | 0.38 | 21 | 3.89×10^-7^ | 40 | 2.08×10^-3^ |
| 3 | 51.6 | 22 | 6.85×10^-5^ | 41 | 1.19×10^-7^ |
| 4 | 924 | 23 | 1.76×10^-5^ | 42 | 3.76×10^-5^ |
| 5 | 133 | 24 | 58.2 | 43 | 5.84×10^-5^ |
| 6 | 2.62×10^-7^ | 25 | 7.81×10^-6^ | 44 | 7.86×10^-4^ |
| 7 | 55.4 | 26 | 1.38 | 45 | 0.02 |
| 8 | 206 | 27 | 0.001 | 46 | 0.31 |
| 9 | 172 | 28 | 5.52 | 47 | 0.60 |
| 10 | 0.03 | 29 | 0.05 | 48 | 0.05 |
| 11 | 4.13×10^-5^ | 30 | 0.12 | 49 | 8.29 |
| 12 | 0.20 | 31 | 6.44×10^-7^ | 50 | 0.06 |
| 13 | 27.6 | 32 | 1.68×10^-5^ | 51 | 2.24 |
| 14 | 0.50 | 33 | 1.31 | 52 | 8.74×10^-8^ |
| 15 | 0.10 | 34 | 1.46 | 53 | 1.71×10^-3^ |
| 16 | 0.01 | 35 | 5.78×10^-3^ | 54 | 1.36 |
| 17 | 233 | 36 | 3.74×10^-4^ | 55 | 0.10 |
| 18 | 1.24 | 37 | 15.4 | 56 | 2.42 |
| 19 | 7.68 | 38 | 3.69×10^-6^ | 57 | 37.2 |

Note: In terms of light and commercial vehicles, the amounts of COD were estimated by the average data base on national level because the outputs of the products are not listed in statistic yearbook of Dalian City.

Reductions of COD discharge by industries are 1.68 Mt in 2015 (i.e., 11.2% of the total emissions in 2010), according to the 12^th^ five year plan of Dalian City. Because reduction target of COD discharge by industries in 2020 has not been released, the target is described as a triangular fuzzy number (i.e., $\tilde{r}$) between the ranges of recent reduction target (i.e., *R* = [10.2%, 12.2%]) with membership of $\mu$, indicating the extent by which *x* belong to fuzzy set $\tilde{r}$ (Equation S38).

$\tilde{r}=(x,\text{ }\mu_{\tilde{r}}(x))|x\in R$ (S38)

An *α-*cut set of $\tilde{r}$ (i.e., $\tilde{r}_{\alpha}$) can be described as an sub-set of $\tilde{r}$ (i.e., $\tilde{r}_{\alpha}=\left\{r\in R|\mu(x)\geq\alpha\right\}$)^18^. The reduction target of COD discharge in multiple α-cut levels could thus indicate its uncertain features. Thus, reductions of COD discharge by industries would be $\left(11.69,\text{ }11.82,\text{ }11.96\right)$ Mt in 2020.

**S2.2.4 Economic parameters**

(1) Profits of industrial products

In this research, the profits of industrial products (i.e., $a_{j}$) are estimated by Equation S39. The prices of wood and compound floorboard are estimated by average market prices. The price of wood floorboard is two times more than compound floorboard. Referred to SBD^19^ and GACC^20^, the profits of industrial products per output are list in Table S9.

$a_{j}=\frac{EP_{j}\times t_{j0}}{\sum_{j=1}^{n'} \left(EP_{j}\times t_{j0}\right)}\times\left(R_{i}-C_{i}\right)$ (S39)

where *R_i_* is the total revenue of related industry from principal business, *C_i_* is the cost of principal business.

**Table S9.** The average economic profits of industries in Dalian City.

| Products | Profit per output | Unit | Products | Profit per output | Unit |
| --- | --- | --- | --- | --- | --- |
| 1 | 7.18×10^6^ | Yuan/10^4^t | 30 | 2.93×10^6^ | Yuan/10^4^t |
| 2 | 6.57×10^6^ | Yuan/100MWh | 31 | 1.56×10^7^ | Yuan/10^4^m^3^ |
| 3 | 7.19×10^7^ | Yuan/10^4^t | 32 | 4.07×10^6^ | Yuan/10^4^ weight boxes |
| 4 | 1.29×10^9^ | Yuan/10^4^t | 33 | 1.77×10^7^ | Yuan/10^4^t |
| 5 | 3.32×10^8^ | Yuan/10^4^t | 34 | 1.98×10^7^ | Yuan/10^4^t |
| 6 | 3.27×10^3^ | Yuan/10^3^L | 35 | 6.50×10^4^ | Yuan/t |
| 7 | 6.90×10^7^ | Yuan/10^4^t | 36 | 4.21×10^4^ | Yuan/t |
| 8 | 3.30×10^8^ | Yuan/10^4^t | 37 | 2.14×10^8^ | Yuan/10^4^m^3^ |
| 9 | 2.76×10^8^ | Yuan/10^4^t | 38 | 51.3×10^6^ | Yuan/10^4^units |
| 10 | 4.28×10^4^ | Yuan/10^4^m | 39 | 3.64×10^6^ | Yuan/t |
| 11 | 6.49×10^5^ | Yuan/10^4^units | 40 | 1.12×10^5^ | Yuan/10^4^KW |
| 12 | 4.28×10^5^ | Yuan/10^4^units | 41 | 6.44×10^4^ | Yuan/unit |
| 13 | 6.05×10^7^ | Yuan/10^4^units | 42 | 2.03×10^5^ | Yuan/t |
| 14 | 5.17×10^6^ | Yuan/10^4^m^2^ | 43 | 3.16×10^5^ | Yuan/10^4^units |
| 15 | 9.82×10^5^ | Yuan/10^4^m^2^ | 44 | 5.70×10^4^ | Yuan/t |
| 16 | 1.17×10^6^ | Yuan/10^4^units | 45 | 1.69×10^6^ | Yuan/t |
| 17 | 7.88×10^7^ | Yuan/10^4^t | 46 | 2.87×10^6^ | Yuan/unit |
| 18 | 5.83×10^6^ | Yuan/10^4^t | 47 | 5.44×10^6^ | Yuan/10^4^tonnage |
| 19 | 2.16×10^7^ | Yuan/10^4^t | 48 | 7.38×10^6^ | Yuan/10^4^units |
| 20 | 1.33×10^5^ | Yuan/t | 49 | 1.12×10^9^ | Yuan/10^4^units |
| 21 | 1.10×10^4^ | Yuan/t | 50 | 8.15×10^6^ | Yuan/10^4^units |
| 22 | 1.93×10^5^ | Yuan/t | 51 | 7.11×10^7^ | Yuan/10^4^units |
| 23 | 4.97×10^5^ | Yuan/t | 52 | 2.57×10^3^ | Yuan/10^4^units |
| 24 | 1.64×10^8^ | Yuan/10^4^t | 53 | 4.50×10^4^ | Yuan/10^4^units |
| 25 | 2.20×10^5^ | Yuan/t | 54 | 4.00×10^7^ | Yuan/10^4^units |
| 26 | 1.35×10^7^ | Yuan/t | 55 | 2.98×10^6^ | Yuan/10^4^units |
| 27 | 3.85×10^4^ | Yuan/10^4^units | 56 | 3.73×10^4^ | Yuan/10^4^units |
| 28 | 1.45×10^8^ | Yuan/10^4^t | 57 | 1.83×10^5^ | Yuan/10^4^units |
| 29 | 1.14×10^6^ | Yuan/10^4^t |  |  |  |

(2) Economic losses of industrial products without water supply

Economic losses of industries without water supply (i.e., the index of $d_{j}$) are estimated by the following equation.

$d_{j}=\frac{EP_{j}\times t_{j0}}{\sum_{j=1}^{n'} \left(EP_{j}\times t_{j0}\right)}\left(1+\theta_{i}\right)\times\left(R_{i}+C_{i}\right)$ (S40)

where *θ_i_* is economic loss rate in *i^th^* industry per unit of water not delivered.

**S2.3 Uncertainty analysis**

(1) Economic profits of industrial products

In this research, the profits of industrial products are estimated by the profits of the related industries from Statistic Bureau of Dalian City^21^. Meanwhile, the profits of industrial products (i.e., vehicles) are estimated by the profits of industrial products from SBS^22^. Thus, the DQI score of vehicles can be described as (4.5, 2.3, 4.3, 2.6, 1.2, 0.9) within level II. Meanwhile, the DQI score of other products can be described as (4.5, 2.3, 4.3, 2.4, 3.2, 1.4) within level IV. The profits of industrial products in 2020 are described in Table S10.

**Table S10.** The average profits of industries in Dalian City ($a^{\pm}$)^*^.

| Products | Profit per output | Products | Profit per output |
| --- | --- | --- | --- |
| 1 | [6.09×10^6^, 8.27×10^6^] | 30 | [2.49×10^6^, 3.37×10^6^] |
| 2 | [5.58×10^6^, 7.56×10^6^] | 31 | [1.32×10^7^, 1.80×10^7^] |
| 3 | [6.10×10^7^, 8.28×10^7^] | 32 | [3.46×10^6^, 4.69×10^6^] |
| 4 | [1.10×10^9^, 1.49×10^9^] | 33 | [1.50×10^7^, 2.04×10^7^] |
| 5 | [2.82×10^8^, 3.82×10^8^] | 34 | [1.68×10^7^, 2.28×10^7^] |
| 6 | [2.80×10^3^, 3.80×10^3^] | 35 | [5.52×10^4^, 7.48×10^4^] |
| 7 | [5.86×10^7^, 7.94×10^7^] | 36 | [3.57×10^4^, 4.85×10^4^] |
| 8 | [2.80×10^8^, 3.80×10^8^] | 37 | [1.82×10^8^, 2.46×10^8^] |
| 9 | [2.34×10^8^, 3.18×10^8^] | 38 | [4.35×10^4^, 5.91×10^4^] |
| 10 | [3.63×10^4^, 4.93×10^4^] | 39 | [3.09×10^6^, 4.19×10^6^] |
| 11 | [5.51×10^5^, 7.47×10^5^] | 40 | [9.51×10^4^, 1.29×10^5^] |
| 12 | [3.63×10^5^, 4.93×10^5^] | 41 | [5.46×10^4^, 7.41×10^4^] |
| 13 | [5.14×10^7^, 6.97×10^7^] | 42 | [1.72×10^5^, 2.34×10^5^] |
| 14 | [4.39×10^6^, 5.95×10^6^] | 43 | [2.68×10^5^, 3.64×10^5^] |
| 15 | [8.34×10^5^, 1.13×10^6^] | 44 | [4.84×10^4^, 6.56×10^4^] |
| 16 | [9.93×10^5^, 1.35×10^6^] | 45 | [1.43×10^6^, 1.95×10^6^] |
| 17 | [6.69×10^7^, 9.07×10^7^] | 46 | [2.44×10^6^, 3.30×10^6^] |
| 18 | [4.95×10^6^, 6.71×10^6^] | 47 | [4.62×10^6^, 6.26×10^6^] |
| 19 | [1.83×10^7^, 2.49×10^7^] | 48 | [6.26×10^6^, 8.50×10^6^] |
| 20 | [1.13×10^5^, 1.53×10^5^] | 49 | [9.51×10^8^, 1.29×10^9^] |
| 21 | [0.93×10^4^, 1.27×10^4^] | 50 | [6.92×10^6^, 9.38×10^6^] |
| 22 | [1.64×10^5^, 2.22×10^5^] | 51 | [6.03×10^7^, 8.18×10^7^] |
| 23 | [4.22×10^5^, 5.72×10^5^] | 52 | [2.18×10^3^, 2.96×10^3^] |
| 24 | [1.39×10^8^, 1.89×10^8^] | 53 | [3.82×10^4^, 5.18×10^4^] |
| 25 | [1.87×10^5^, 2.53×10^5^] | 54 | [3.40×10^7^, 4.61×10^7^] |
| 26 | [1.15×10^7^, 1.55×10^7^] | 55 | [2.53×10^6^, 3.43×10^6^] |
| 27 | [3.27×10^4^, 4.43×10^4^] | 56 | [2.01×10^4^, 5.46×10^4^] |
| 28 | [1.23×10^8^, 1.67×10^8^] | 57 | [9.85×10^4^, 2.67×10^5^] |
| 29 | [9.67×10^5^, 1.31×10^6^] |  | |

*Note: The units of average profits in industrial products are the same as the units in Table 8.

(2) Water consumption of industrial products

In this research, water consumption of industrial products is referred to the standards of water consumption quotas from multiple provinces (e.g., Fujian, Yunnan and Liaoning). Uncertainty features of these indexes (i.e., $t^{\pm}$) are described in Table S11.

**Table S11.** Water consumption quotas of main industrial products in Dalian City.

| Industrial Products | DQI | Level | Quotas | Unit | Industrial Products | DQI | Level | Quotas | Unit |
| --- | --- | --- | --- | --- | --- | --- | --- | --- | --- |
| 1 | (4.2, 1.9, 4.1, 3.2, 4.1, 3.5) | V | [0.94, 1.06] | m^3^/t | 30 | (4.2, 1.9, 4.1, 3.2, 4.1, 3.5) | V | [0.14, 1.59] | m^3^/t |
| 2 | (4.2, 1.9, 4.1, 3.2, 4.1, 3.5) | V | [2.26, 3.39] | m^3^/MWh | 31 | (4.2, 1.9, 4.1, 3.2, 4.1, 3.5) | V | [0.94, 2.12] | m^3^/m^3^ |
| 3 | (4.2, 1.9, 4.1, 3.2, 4.1, 3.5) | V | [0.38, 1.27] | m^3^/t | 32 | (4.2, 1.9, 4.1, 3.2, 4.1, 3.5) | V | [0.38, 0.64] | m^3^/t |
| 4 | (4.2, 1.9, 4.1, 3.2, 4.1, 3.5) | V | [4.70, 5.83] |  | 33 | (4.2, 1.9, 4.1, 3.2, 4.1, 3.5) | V | [4.23, 7.42] |  |
| 5 | (4.2, 1.9, 4.1, 3.2, 4.1, 3.5) | V | [4.70, 16.96] |  | 34 | (4.2, 1.9, 4.1, 2.9, 1.2, 1.5) | V | [1.88, 4.24] |  |
| 6 | (4.2, 1.9, 4.1, 3.2, 4.1, 3.5) | V | [2.90, 4.01] | m^3^/10^3^L | 35 | (4.2, 1.9, 4.1, 3.2, 4.1, 3.5) | V | [6.18, 6.98] |  |
| 7 | (4.2, 1.9, 4.1, 3.2, 4.1, 3.5) | V | [1.88, 2.97] | m^3^/t | 36 | (4.2, 1.9, 4.1, 1.2, 1.2, 1.5) | II | [4.85, 13.15] |  |
| 8 | (4.2, 1.9, 4.1, 3.2, 4.1, 3.5) | V | [17.85, 20.15] |  | 37 | (4.2, 1.9, 4.1, 1.2, 1.2, 1.5) | II | [7.00, 19.00] |  |
| 9 | (4.2, 1.9, 4.1, 3.2, 4.1, 3.5) | V | [18.79, 21.21] |  | 38 | (4.2, 1.9, 4.1, 3.2, 4.1, 3.5) | V | [12.22, 13.78] | m^3^/100 units |
| 10 | (4.2, 1.9, 4.1, 3.2, 4.1, 3.5) | V | [1.22, 1.38] | m^3^/100m | 39 | (4.2, 1.9, 4.1, 3.2, 4.1, 3.5) | V | [56.38, 63.61] | m^3^/t |
| 11 | (4.2, 1.9, 4.1, 3.2, 4.1, 3.5) | V | [84.57, 95.42] | m^3^/10^4^ units | 40 | (4.2, 1.9, 4.1, 3.2, 4.1, 3.5) | V | [921, 1378] | m^3^/10^4^KW |
| 12 | (4.2, 1.9, 4.1, 3.2, 4.1, 3.5) | V | [44.18, 49.83] |  | 41 | (4.2, 1.9, 4.1, 3.2, 4.1, 3.5) | V | [117, 133] | m^3^/unit |
| 13 | (4.2, 1.9, 4.1, 2.9, 1.2, 1.5) | V | [2115, 2385] |  | 42 | (4.2, 1.9, 4.1, 3.2, 4.1, 3.5) | V | [12.21, 13.78] | m^3^/t |
| 14 | (4.2, 1.9, 4.1, 1.2, 1.2, 1.5) | II | [3.23, 8.77] | m^3^/km^2^ | 43 | (4.2, 1.9, 4.1, 3.2, 4.1, 3.5) | V | [32.89, 37.11] | m^3^/10^4^ units |
| 15 | (4.2, 1.9, 4.1, 1.2, 1.2, 1.5) | II | [10.77, 29.22] |  | 44 | (4.2, 1.9, 4.1, 2.6, 1.2, 1.5) | V | [22.55, 25.45] | m^3^/t |
| 16 | (4.2, 1.9, 4.1, 3.2, 4.1, 3.5) | V | [0.19, 0.53] | m^3^/unit | 45 | (4.2, 1.9, 4.1, 3.2, 4.1, 3.5) | V | [22.55, 25.44] |  |
| 17 | (4.2, 1.9, 4.1, 3.2, 4.1, 3.5) | V | [18.80, 31.81] | m^3^/t | 46 | (4.2, 1.9, 4.1, 3.2, 4.1, 3.5) | V | [3289, 3711] | m^3^/unit |
| 18 | (4.2, 1.9, 4.1, 3.2, 4.1, 3.5) | V | [0.55, 0.61] |  | 47 | (4.2, 1.9, 4.1, 2.6, 1.2, 1.5) | V | [65.78, 116.63] | m^3^/ tonnage |
| 19 | (4.2, 1.9, 4.1, 3.2, 4.1, 3.5) | V | [14.09, 23.33] |  | 48 | (4.2, 1.9, 4.1, 3.2, 4.1, 3.5) | V | [0.47, 0.53] | m^3^/unit |
| 20 | (4.2, 1.9, 4.1, 3.2, 4.1, 3.5) | V | [3.10, 4.77] |  | 49 | (4.2, 1.9, 4.1, 3.2, 4.1, 3.5) | V | [0.47, 0.53] |  |
| 21 | (4.2, 1.9, 4.1, 3.2, 4.1, 3.5) | V | [12.22, 28.63] |  | 50 | (4.2, 1.9, 4.1, 3.2, 4.1, 3.5) | V | [43.23, 48.78] |  |
| 22 | (4.2, 1.9, 4.1, 3.2, 4.1, 3.5) | V | [46.98, 103.90] |  | 51 | (4.2, 1.9, 4.1, 1.2, 1.2, 1.5) | II | [5.92, 16.08] |  |
| 23 | (4.2, 1.9, 4.1, 3.2, 4.1, 3.5) | V | [1.88, 10.60] |  | 52 | (4.2, 1.9, 4.1, 3.2, 4.1, 3.5) | V | [0.19, 2.54] |  |
| 24 | (4.2, 1.9, 4.1, 3.2, 4.1, 3.5) | V | [13.63, 17.50] |  | 53 | (4.2, 1.9, 4.1, 3.2, 4.1, 3.5) | V | [1.32, 1.80] | m^3^/unit |
| 25 | (4.2, 1.9, 4.1, 3.2, 4.1, 3.5) | V | [2.82, 3.18] |  | 54 | (4.2, 1.9, 4.1, 3.2, 4.1, 3.5) | V | [0.05, 0.05] |  |
| 26 | (4.2, 1.9, 4.1, 3.2, 4.1, 3.5) | V | [188, 212] |  | 55 | (4.2, 1.9, 4.1, 1.2, 1.2, 1.5) | II | [97, 263] | m^3^/10^4^units |
| 27 | (4.2, 1.9, 4.1, 3.2, 4.1, 3.5) | V | [160, 270] |  | 56 | (4.2, 1.9, 4.1, 3.2, 4.1, 3.5) | V | [4.23, 4.77] | m^3^/unit |
| 28 | (4.2, 1.9, 4.1, 3.2, 4.1, 3.5) | V | [2.82, 8.48] |  | 57 | (4.2, 1.9, 4.1, 3.2, 4.1, 3.5) | V | [4.23, 4.77] |  |
| 29 | (4.2, 1.9, 4.1, 2.4, 1.2, 1.5) | II | [0.11, 0.29] |  |  |  |  |  |  |

(3) The minimum growth rate of industrial production

The minimum growth rates of industrial production in Dalian City are estimated by the related plans from PRIMI^23^ and Dalian Municipal Government^24^. In detail, the growth rates of ships, vehicles, and electrical appliances in 2020 are estimated based on Planning Research Institute for Machinery Industry (PRIMI)^23^; the growth rates of crude oil, medicines, and rolled steel are referred to DMG^24^. Thus, the DQI scores of equipment products can be described as (4.6, 1.8, 4.5, 2.1, 4.8, 4.6) in level III, and the DQI scores of other preferential developmental products can be described as (4.6, 1.8, 4.5, 2.8, 4.8, 4.6) in level III. Uncertainty features of minimum growth rate of industrial products ($\varepsilon^{\pm}$) are described in Table S12.

**Table S12.** The plan for dominate industrial products in Dalian City.

| Industrial Products | Base Year (2010) | Planning year (2020) | Unit |
| --- | --- | --- | --- |
| Chemical fiber | 2.4 | [6.0, 12.4] | 10^4^ t |
| Yarn | 2.3 | [5.8, 11.9] | 10^4^ t |
| Cloth | 7.5 | [18.8, 38.8] | 10^6^ m |
| Garments | 123.5 | [310, 638] | 10^6^ units |
| Crude oil processed | 26.5 | [26.1, 53.9] | 10^6^ t |
| Traditional Chinese medicines | 164 | [412, 847] | t |
| Rolled steel | 183.8 | [461, 950] | 10^4^ t |
| Meta-cutting machine | 52 | [123, 253] | 10^3^ units |
| Rolling contact bearing | 147.5 | [283, 583] | 10^6^ units |
| Rail vehicles | 589 | [1410, 2910] | Unit |
| Civilian-used steel ships | 6.4 | [15.2, 31.4] | 10^6^ tonnage |
| Household refrigerators | 44.6 | [83.5, 172] | 10^4^ units |
| Commercial refrigerators | 24.4 | [45.7, 94.1] |
| Air conditioners | 163.8 | [306, 632] |
| Printer | 85.1 | [159, 328] |
| Color television sets | 247 | [462, 953] |
| video disc player | 298.7 | [559, 1150] |
| Light vehicles | - | [0.6, 1.2] |
| Commercial vehicles | - | [0.3, 0.6] |

The index of minimum annual growth of regional GDP is predicted based on the related indexes in the12^th^ Five year planning of Dalian City (i.e., 13%). Thus, the DQI of the index could be described as (4.8, 2.5, 4.5, 2.1, 4.5, 2.8) within level V. Thus, the minimum annual growth rate of GDP in Dalian City between 2015 and 2020 would be [7%, 13.79%].

**S2.4 Results**

**Table S13.** The solutions for output of industrial products.

| *α*-cut | 1 | 0.8 | 0.6 | 0.4 | 0.2 |
| --- | --- | --- | --- | --- | --- |
| S1 | | | | | |
| 1 | [56.43, 79.69] | [156.10, 243.01] | [89.67, 150.02] | [77.05, 156.03] | [42.20, 150.74] |
| 2 | [83.59, 513.10] | [216.22, 678.54] | [164.53, 306.04] | [444.18, 697.92] | [8.35, 254.06] |
| 3 | [16.76, 17.01] | [28.59, 51.96] | [14.06, 15.58] | [109.87, 115.62] | [0.51, 1.14] |
| 4 | [0.18, 4.10] | [1.18, 1.23] | [4.21, 4.73] | [0.04, 0.31] | [1.07, 9.20] |
| 5 | [18.44, 18.75] | [2.31, 2.67] | [17.88, 17.94] | [3.75, 10.50] | [20.56, 25.05] |
| 6 | [203744, 261844] | [203947, 769944] | [695445, 1428531] | [373444, 1681834] | [700997, 890884] |
| 7 | [20.03, 45.69] | [7.93, 19.85] | [0.004, 8.80] | [20.78, 56.38] | [27.79, 30.58] |
| 8 | [1.48, 8.47] | [3.38, 6.00] | [2.35, 3.68] | [0.36, 8.86] | [3.55, 3.76] |
| 9 | [0.10, 0.51] | [9.12, 11.39] | [2.95, 3.30] | [2.55, 6.52] | [2.68, 2.70] |
| 10 | [1721, 2882] | [195.04, 1177] | [753.57, 848.87] | [956.14, 1214] | [315.98, 1529] |
| 11 | [27934, 28708] | [21288, 32645] | [21452, 42404] | [20669, 34639] | [22176, 32513] |
| 12 | [257.29, 520.45] | [147.90, 301.79] | [359.36, 561.32] | [314.35, 400.96] | [153.91, 163.94] |
| 13 | [63.69, 66.25] | [23.48, 44.26] | [1.74, 53.50] | [4.48, 52.27] | [26.94, 59.49] |
| 14 | [137.35, 229.87] | [85.19, 382.01] | [311.77, 554.19] | [324.64, 522.31] | [62.12, 394.15] |
| 15 | [211.48, 234.38] | [9.58, 33.38] | [56.13, 148.64] | [130.58, 271.08] | [135.56, 162.28] |
| 16 | [2536, 3745] | [806.80, 4250.92] | [2363, 2649] | [1959, 3286] | [113.73, 267.18] |
| 17 | [12.24, 12.36] | [3.38, 3.97] | [3.76, 5.37] | [0.03, 0.15] | [6.63, 22.26] |
| 18 | [421.68, 1980.18] | [439.07, 984.68] | [43.84, 395.60] | [782.08, 951.48] | [185.12, 1504] |
| 19 | [14.74, 22.65] | [23.28, 36.11] | [0.16, 7.44] | [18.04, 23.98] | [2.44, 21.23] |
| 20 | [32464, 67889] | [6990, 58948] | [28950, 45197] | [16042, 40962] | [24260, 43883] |
| 21 | [38557, 40427] | [5155, 130777] | [79063, 224719] | [27197, 94547] | [7566, 26627] |
| 22 | [9417, 29527] | [1567, 4736] | [9706, 18157] | [751.75, 2989.75] | [11779, 23275] |
| 23 | [68678, 127029] | [2159, 4742] | [25974, 44770] | [5942 116100] | [6874, 27912] |
| 24 | [65.48, 127.21] | [9.30, 30.22] | [99.45, 99.54] | [5.70, 41.82] | [13.30, 19.82] |
| 25 | [30920, 35530] | [21391, 25511] | [11352, 37005] | [7829, 31356] | [71200, 74836] |
| 26 | [42.01, 108.06] | [106.93, 571.86] | [226.24, 631.82] | [161.01, 581.65] | [258.48, 494.92] |
| 27 | [390.27, 1087.07] | [822.59, 1834.59] | [42.50, 77.57] | [92.77, 2215.04] | [49.93, 159.07] |
| 28 | [32.24, 70.98] | [25.63, 79.78] | [99.16, 125.13] | [23.92, 26.07] | [38.73, 114.78] |
| 29 | [24.55, 137.55] | [289.84, 2442.15] | [980.91, 3169.71] | [315.45, 788.75] | [169.75, 2585.65] |
| 30 | [663.24, 2657.14] | [422.96, 1596.94] | [366.89, 3132.63] | [2482, 2509] | [121.00, 1288.04] |
| 31 | [56.91, 84.52] | [458.11, 928.75] | [274.47, 471.53] | [34.01, 551.86] | [440.48, 852.37] |
| 32 | [2.48, 506.38] | [801.21, 1666.72] | [229.50, 1195.54] | [1159, 1520] | [81.41, 1083.28] |
| 33 | [151.75, 183.01] | [58.24, 149.73] | [42.61, 222.53] | [75.98, 278.22] | [0.66, 11.44] |
| 34 | [20451, 21297] | [17159, 23890] | [21391, 21737] | [19071, 20935] | [17185, 17468] |
| 35 | [8111, 10231] | [3069, 5176] | [3099, 8093] | [7360, 12367] | [1110, 15906] |
| 36 | [4166, 15917] | [12833, 14710] | [5245, 6058] | [2075, 16293] | [3338, 9490] |
| 37 | [48.21, 69.85] | [3.21, 4.31] | [26.97, 40.73] | [3.44, 5.94] | [38.46, 39.02] |
| 38 | [1075, 2621] | [2648, 3548] | [134.14, 209.72] | [575.42, 2816] | [570, 2554] |
| 39 | [2683, 3466] | [1612, 1729] | [2436, 3851] | [63.13, 153.33] | [1649, 3584] |
| 40 | [10638, 12742] | [1372, 1671] | [3189, 12097] | [2248, 14380] | [234.89, 1350.64] |
| 41 | [138045, 271743] | [800, 263784] | [9807, 225788] | [40295, 148715] | [44630, 69995] |
| 42 | [6668, 15468] | [51156, 76528] | [90362, 402351] | [51018, 408611] | [142742, 323788] |
| 43 | [10548, 27223] | [1417, 55548] | [19221, 33641] | [32378, 33269] | [6928, 26771] |
| 44 | [448991, 515501] | [597884, 634920] | [249313, 455218] | [302881, 306071] | [243244, 288521] |
| 45 | [7756, 8614] | [40095, 40526] | [25714, 28989] | [18469, 20670] | [29898, 33325] |
| 46 | [1573, 1719] | [25752, 26625] | [805, 6273] | [26537, 27464] | [23757, 23899] |
| 47 | [5.06, 129.06] | [8.48, 261.56] | [0.48, 275.47] | [4.26, 147.36] | [7.24, 594.56] |
| 48 | [41.27, 88.22] | [51.54, 80.79] | [54.28, 112.38] | [47.49, 118.69] | [77.84, 101.47] |
| 49 | [95.29, 97.60] | [31.22, 33.99] | [79.38, 80.70] | [60.82, 75.03] | [96.51, 97.94] |
| 50 | [43.89, 302.49] | [43.10, 435.89] | [7.34, 8.57] | [2.56, 52.76] | [0.33, 79.59] |
| 51 | [101.84, 340.06] | [10.43, 24.47] | [335.99, 375.32] | [46.27, 86.54] | [92.93, 175.24] |
| 52 | [396872, 422372] | [522997, 670872] | [108253, 170324] | [517472, 1158332] | [630301, 631172] |
| 53 | [16.10, 21.80] | [18.93, 31.64] | [23.39, 26.33] | [0.01, 0.38] | [4.73, 18.04] |
| 54 | [14.52, 21.72] | [588.41, 895.12] | [443.50, 1020.79] | [642.02, 1045.33] | [139.20, 262.30] |
| 55 | [691.29, 874.74] | [328.25, 565.71] | [464.45, 537.29] | [26.01, 153.41] | [64.82, 607.86] |
| 56 | [0.06, 0.98] | [0.22, 0.73] | [0.45, 0.57] | [0.13, 0.43] | [0.74, 0.94] |
| 57 | [0.14, 0.26] | [0.004, 0.62] | [0.31, 0.68] | [0.29, 0.36] | [0.02, 0.37] |
| S2 | | | | | |
| 1 | [110.05, 136.30] | [11.74, 28.13] | [16.37, 167.56] | [14.28, 28.81] | [7.98, 17.07] |
| 2 | [313.91, 363.79] | [309.60, 698.36] | [61.33, 64.10] | [286.15, 523.01] | [101.36, 456.90] |
| 3 | [2.02, 2.28] | [0.67, 1.07] | [1.01, 2.91] | [35.04, 51.30] | [11.16, 17.62] |
| 4 | [6.52, 9.96] | [4.76, 13.55] | [0.59, 1.61] | [3.41, 4.16] | [15.67, 17.64] |
| 5 | [2.34, 4.72] | [32.09, 42.82] | [8.05, 22.36] | [4.34, 6.49] | [12.76, 13.47] |
| 6 | [98344, 149544] | [94266, 115744] | [1315092, 1813290] | [346344, 1386574] | [318581, 830244] |
| 7 | [51.63, 83.77] | [3.14, 4.94] | [11.00, 50.44] | [2.30, 8.36] | [83.33, 101.40] |
| 8 | [0.13, 2.73] | [8.64 9.29] | [1.37, 7.06] | [0.01, 0.19] | [0.25, 0.97] |
| 9 | [3.10, 11.92] | [0.21, 0.54] | [0.01, 5.10] | [0.05, 2.47] | [3.42, 7.71] |
| 10 | [15.24, 20.34] | [1629, 3424] | [1354, 2803] | [1082, 1217] | [144, 2095] |
| 11 | [18510, 27621] | [31136, 61692] | [34048, 43886] | [26931, 44065] | [12866, 21793] |
| 12 | [16.81, 30.39] | [252.81, 521.84] | [289.78, 352.87] | [19.79, 531.34] | [291.33, 508.65] |
| 13 | [19.78, 37.96] | [3.56, 36.80] | [3.63, 15.82] | [12.94, 21.26] | [0.95, 13.48] |
| 14 | [117.53, 186.84] | [8.01, 107.29] | [510.56, 536.75] | [53.51, 462.86 | [184.66, 477.50 |
| 15 | [626.83, 687.33] | [72.48, 1250.72] | [208.83, 236.80] | [120.18, 1101.33] | [196.92, 415.18] |
| 16 | [10.68, 154.28] | [810.24, 1344.88] | [329.90, 1220.03] | [3085.28, 4263.60] | [1205.12, 1440.58] |
| 17 | [0.84, 1.44] | [0.93, 1.93] | [23.27, 25.29] | [6.23, 49.59] | [2.77, 5.52] |
| 18 | [46.28, 617.78] | [238.50, 515.88] | [1437.06, 2239.33] | [475.78, 2269.78] | [149.20, 253.18] |
| 19 | [0.66, 20.69] | [4.90, 7.44] | [9.70, 21.75] | [1.74, 7.19] | [0.99, 8.22] |
| 20 | [1541, 4415] | [15285, 35133] | [1989, 4794] | [3950, 19655] | [67155, 70084] |
| 21 | [53797, 242712] | [54920, 212861] | [182948, 266350] | [27957, 29477] | [845, 253408 |
| 22 | [1135, 4702] | [1280, 22168] | [248, 10446] | [16053, 19833] | 15993, 18516] |
| 23 | [12372, 86631] | [45982, 97285] | [33690, 46602] | [102294, 127391] | [137668 147765] |
| 24 | [10.35, 45.44] | [11.49, 26.77] | [32.79, 134.87] | [21.69, 40.49] | [11.26, 42.42] |
| 25 | [24624, 25405] | [9172, 14899] | [31410, 76421] | [3138, 65216] | [24940, 70752] |
| 26 | [266.26, 715.48] | [247.94, 735.32] | [58.76, 73.00] | [441.32, 570.46] | [137.64, 694.43] |
| 27 | [1138, 1177] | [482.40, 1089] | [150.22, 534.90] | [462.57, 787.87] | [18.90, 283.17] |
| 28 | [48.14, 62.87] | [20.62, 55.15] | [83.22, 121.40] | [14.23, 68.37] | [60.21, 93.78] |
| 29 | [1724, 1738] | [102, 144] | [2167, 2291] | [1165, 3230] | [73.73, 916.25] |
| 30 | [1786, 4023] | [1683, 2180] | [1467, 2427] | [3277, 3511] | [442.08, 2032] |
| 31 | [622.18, 747.33] | [1.24, 38.81] | [772.58, 1036.90] | [689.14, 845.70] | [2.47, 97.44] |
| 32 | [1905, 2067] | [383, 1127] | [1949, 2293] | [443, 2275] | [1650, 2198] |
| 33 | [173.33, 198.68] | [144.73, 194.34] | [73.00, 251.29] | [252.73, 307.44] | [77.40 339.63] |
| 34 | [12585, 12751] | [15654, 16609] | [13936, 14767] | [9430, 9952] | [11742, 12400] |
| 35 | [3464, 5748] | [3331, 7406] | [8190, 8269] | [7166, 8842] | [8793, 13724] |
| 36 | [2692, 25581] | [18785, 21834] | [10244, 24904] | [1112, 22322] | [18028, 24721] |
| 37 | [49.72, 66.84] | [1.15, 2.04] | [26.84, 72.50] | [13.46, 56.20] | [21.04 44.05] |
| 38 | [1275, 6699] | [947, 1056] | [2553, 3950] | [1993, 2759] | [754, 2031] |
| 39 | [474.83, 671.43] | [132.16, 424.73] | [135.80, 138.48] | [202.03, 230.53] | [2073.88, 3305.07] |
| 40 | [1904, 11074] | [4191, 10810] | [3288, 15703] | [7550, 11445] | [989, 1751] |
| 41 | [125295, 172394] | [58521, 63535] | [164147, 197869] | [230172, 242501] | [76685 214957] |
| 42 | [19748, 399915] | [259548, 266138] | [151947, 340933] | [115508, 137308] | [60117, 393675] |
| 43 | [22954, 37153] | [37186, 50565] | [23929, 41691] | [34514, 42466] | [20416, 23608] |
| 44 | [289941, 345771] | [352323, 369161] | [80345, 167362] | [256271, 451781] | [199475, 200851] |
| 45 | [220.07, 377.07] | [18654, 21637] | [15773, 18160] | [38306, 39262] | [25703 38582] |
| 46 | [1602, 3355] | [8529, 10526] | [11438, 22000] | [22577, 27840] | [3309, 6279] |
| 47 | [161.66, 441.96] | [163.23, 608.56] | [146.18, 255.99] | [216.36 674.06] | [251.50, 844.76] |
| 48 | [10.39, 129.95] | [25.70, 143.83] | [15.15, 52.56] | [5.36, 95.86] | [103.05, 187.98] |
| 49 | [88.90, 94.00] | [6.59, 9.98] | [67.75, 68.08] | [43.34, 45.70] | [58.14 59.61] |
| 50 | [11.31, 268.16] | [38.02, 101.75] | [126.51, 551.98] | [68.51, 80.58] | [162.86, 270.13] |
| 51 | [2.66, 12.66] | [47.02, 75.95] | [13.09, 48.07] | [174.42, 338.75] | [18.94, 22.92] |
| 52 | [14272, 107272] | [798330, 1116892] | [217024, 449094] | [458072, 1758906] | [163651, 813372] |
| 53 | [32.52, 34.06] | [38.83, 39.54] | [0.12, 4.20] | [15.19, 19.98] | [3.04 5.82] |
| 54 | [126.20, 196.83] | [83.60, 88.92] | [200.23, 538.15] | [661.18, 810.97] | [128.72, 703.09] |
| 55 | [14.41, 129.31] | [543.91, 609.75] | [282.13, 1078] | [16.21, 16.61] | [299.57, 1182] |
| 56 | [0.22, 0.85] | [0.37, 1.18] | [0.54, 0.66] | [0.21, 0.36] | [0.07, 0.45] |
| 57 | [0.10, 0.23] | [0.06, 0.32] | [0.07, 0.08] | [0.44, 0.48] | [0.31, 0.69] |
| S3 | | | | | |
| 1 | [34.09, 43.94] | [85.21, 89.43] | [52.53, 69.07] | [75.44, 28.52] | [26.93, 64.56] |
| 2 | [156.07, 573.99] | [222.54, 439.07] | [28.47, 197.40] | [521.08, 692.41] | [284.48, 641.70] |
| 3 | [8.17, 52.28] | [65.01, 89.16] | [1.77, 22.09] | [24.16, 40.85] | [12.99, 20.70] |
| 4 | [0.26, 1.74] | [0.32, 1.04] | [1.38, 3.73] | [3.18, 5.56] | [1.44, 4.09] |
| 5 | [9.37, 9.50] | [22.73, 30.95] | [5.09, 12.65] | [0.98, 12.11] | [9.09, 12.12] |
| 6 | [81044, 376044] | [21782, 192744] | [400400, 1114584] | [171744, 584744] | [1072790, 1317220] |
| 7 | [4.43, 12.04] | [8.75, 95.36] | [45.05, 96.94] | [41.39, 53.59] | [0.72, 1.13] |
| 8 | [2.52, 4.78] | [3.65, 3.79] | [7.37, 10.72] | [0.29, 2.15] | [1.67, 1.80] |
| 9 | [3.57, 4.25] | [7.45, 9.16] | [1.59, 1.78] | [3.53, 3.60] | [0.65, 1.69] |
| 10 | [2662, 3476] | [2062, 3687] | [229, 365] | [1064, 2422] | [1281, 2691] |
| 11 | [7752, 15477] | [1502, 12484] | [46975, 51852] | [26274, 50621] | [17610, 34891] |
| 12 | [514.82, 640.10] | [339.54, 477.20] | [5.07, 7.23] | [17.68, 20.39] | [319.25, 658.98] |
| 13 | [0.82, 1.98] | [0.41, 54.69] | [27.41, 27.84] | [7.67, 9.44] | [2.16, 22.34] |
| 14 | [297.91, 547.30] | [62.34, 257.11] | [179.96, 446.03] | [368.21, 375.38] | [26.10, 349.77] |
| 15 | [11.18, 30.98] | [12.24, 196.98] | [386.04, 1050.66] | [1273.82, 1280.46] | [16.29, 281.12] |
| 16 | [2163, 2345] | [737, 1634] | [912, 2353] | [367, 596] | [1718, 2851] |
| 17 | [34.09, 54.88] | [1.04, 1.52] | [20.44, 21.59] | [8.81, 24.17] | [20.80, 43.11] |
| 18 | [474, 720] | [5822, 6172] | [4927, 5072] | [3591, 4898] | [1318, 2852] |
| 19 | [5.39, 9.98] | [3.89, 4.41] | [1.46, 6.84] | [5.50, 15.42] | [6.22, 9.44] |
| 20 | [30067, 34272] | [5740, 69191] | [5202, 47974] | [20849, 74713] | [628, 1444] |
| 21 | [95367, 115297] | [175558, 280545] | [134343, 145747] | [168777, 246850] | [69350, 268792] |
| 22 | [11101, 11149] | [12645, 18747] | [1417, 3494] | [13971, 33084] | [1488, 25762] |
| 23 | [14872, 49102] | [16310, 65621] | [45758, 148993] | [5002, 90959] | [9943, 21038] |
| 24 | [63.46, 86.64] | [4.76, 29.71] | [43.00, 78.38] | [1.01, 2.31] | [55.91, 130.23] |
| 25 | [7167, 52380] | [16585, 25804] | [9316, 70216] | [39722, 77008] | [30906, 50202] |
| 26 | [512.42, 847.20] | [279.73, 381.15] | [31.31, 36.41] | [104.69, 148.74] | [200.14, 593.56] |
| 27 | [785.37, 825.67] | [1853,  2163.17] | [828.65, 1811] | [125.37, 417.57] | [721.80, 1629] |
| 28 | [92.02, 105.87] | [79.29, 111.96] | [10.87, 11.17] | [11.28, 35.00] | [26.60, 71.14] |
| 29 | [191.85, 676.25] | [56.49, 1239] | [2160, 2823] | [457.55, 495.75] | [1871, 2647] |
| 30 | [228.04, 1525] | [1625, 4002] | [289.35, 564.24] | [344.44, 1152] | [1303, 1688] |
| 31 | [337.73, 937.67] | [72.78, 772.49] | [241.43, 241.52] | [413.56, 797.82] | [18.09, 564.90] |
| 32 | [772.08, 1500.68] | [474.20, 1708] | [1980, 2113] | [388.38, 1317] | [682.58, 2011] |
| 33 | [5.17, 12.66] | [194.65, 228.75] | [115.76, 196.96] | [0.29, 1.38] | [258.66, 347.32] |
| 34 | [13586, 13743] | [12997, 13112] | [16880, 16924] | [14459, 15816] | [9195, 9757] |
| 35 | [1324, 1601] | [73.54, 10183] | [1050, 3901] | [1316, 12045] | [751.94, 1672] |
| 36 | [5406, 13881] | [13424, 23275.37] | [11.90, 3269] | [7291.37, 14363] | [24157, 28078] |
| 37 | [24.05, 48.13] | [0.88, 1.15] | [14.40, 40.47] | [30.91, 67.33] | [24.52, 43.44] |
| 38 | [143.92, 220.82] | [3491, 4558] | [6381, 7207] | [2006, 5078] | [5340, 5957] |
| 39 | [521.93, 789.73] | [580.92, 1320] | [1898, 2572] | [411.53, 1960] | [270.32, 868.73] |
| 40 | [4664, 13212] | [10384, 14578] | [1834, 8553] | [222.64, 15031] | [5212, 13442] |
| 41 | [175938, 260476] | [107461, 256752] | [9063, 132335] | [69235, 150965] | [123597, 134187] |
| 42 | [85638, 304648] | [356456, 383822] | [141232, 241158] | [144588, 215258] | [324236, 332469] |
| 43 | [28372, 31036] | [29921, 42278] | [13524, 56357] | [10748, 25091] | [29036, 39483] |
| 44 | [5081, 71751] | [143041, 296231] | [42225, 293351] | [422291, 512171] | [265878, 278585] |
| 45 | [34538, 38310] | [22910, 30062] | [8413, 8674] | [16349, 18980] | [33796, 39200] |
| 46 | [11869, 14959] | [6824, 15127] | [5534, 6362] | [7346, 8577] | [10270, 12674] |
| 47 | [193.06, 385.36] | [53.24, 561.56] | [31.77, 272.76] | [183.26, 404.76] | [141.35, 526.98] |
| 48 | [41.62, 139.45] | [166.58, 182.32] | [4.48, 152.15] | [6.96, 7.81] | [0.89, 4.96] |
| 49 | [65.92, 74.65] | [25.32, 42.70] | [80.04, 87.54] | [35.65, 53.74] | [36.76, 55.61] |
| 50 | [40.27, 65.48] | [85.67, 98.85] | [107.79, 492.70] | [122.73, 180.65] | [66.26, 177.35] |
| 51 | [193.74, 270.81] | [234.05, 319.84] | [23.12, 226.14] | [55.08, 82.64] | [215.31, 347.79] |
| 52 | [766972, 1222912] | [116957, 129472] | [38237, 889202] | [122472, 1427832] | [621786, 869901] |
| 53 | [12.17, 36.42] | [32.63, 33.66] | [12.69, 14.34] | [8.98, 31.48] | [8.08, 8.23] |
| 54 | [293.64, 444.61] | [445.65, 547.17] | [150.31, 399.16] | [664.47, 701.33] | [995, 1058] |
| 55 | [440.05, 528.18] | [167.48, 421.54] | [[544.53, 733.26] | [52.01, 91.51] | [748.70, 839.33] |
| 56 | [0.49, 0.83] | [0.50, 0.57] | [0.15, 0.19] | [0.64, 0.77] | [0.30, 0.98] |
| 57 | [0.31, 0.42] | [0.38, 0.39] | [0.19, 0.31] | [0.32, 0.65] | [0.12, 0.68] |

**References**

1. May, J. & Brennan, D. Application of data quality assessment methods to an LCA of electricity generation. *International Journal of Life Cycle Assessment* **8**, 215-225 (2003).

2. Lewandowska, A., Foltynowicz, Z. & Podlesny, A. Comparative LCA of industrial objects - Part 1: LCA data quality assurance - Sensitivity analysis and pedigree matrix. *International Journal of Life Cycle Assessment* **9**, 86-89 (2004).

3. Wang, E. & Shen, Z. A hybrid Data Quality Indicator and statistical method for improving uncertainty analysis in LCA of complex system - application to the whole-building embodied energy analysis. *Journal of Cleaner Production* **43**, 166-173 (2013).

4. Jahani, E., Muhanna, R. L., Shayanfar, M. A. & Barkhordari, M. A. Reliability Assessment with Fuzzy Random Variables Using Interval Monte Carlo Simulation. *Computer-Aided Civil and Infrastructure Engineering* **29**, 208-220 (2014).

5. Minasny, B. & McBratney, A. B. A conditioned Latin hypercube method for sampling in the presence of ancillary information. *Computers & Geosciences* **32**, 1378-1388 (2006).

6. Shin, P. S., Woo, S. H., Zhang, Y. & Koh, C. S. An Application of Latin Hypercube Sampling Strategy for Cogging Torque Reduction of Large-Scale Permanent Magnet Motor. *IEEE Transactions on Magnetics* **44**, 4421-4424 (2008).

7. Huang, G. H., Baetz, B. W. & Patry, G. G. Grey integer programming: an application to waste management planning under uncertainty. *European Journal of Operational Research* **83**, 594-620 (1995).

8. Cai, Y. P., Huang, G. H., Tan, Q. & Yang, Z. F. An integrated approach for climate-change impact analysis and adaptation planning under multi-level uncertainties. Part I: Methodology. *Renewable and Sustainable Energy Reviews* **15**, 2779-2790 (2011).

9. Han, Y., Huang, Y.-F., Wang, G.-Q. & Maqsood, I. A Multi-objective Linear Programming Model with Interval Parameters for Water Resources Allocation in Dalian City. *Water Resources Management* **25**, 449-463 (2011).

10. Cai, Y., Yue, W., Xu, L., Yang, Z. & Rong, Q. Sustainable urban water resources management considering life-cycle environmental impacts of water utilization under uncertainty. *Resources, Conservation and Recycling* **108**, 21-40 (2016).

11. Dalian Water Affairs Bureau (DWAB). *Plans for Dalian water resources management*. (Dalian Water Affairs Bureau, 2012).

12. Administration of quality and technology supervision of Guangdong Province (AQTSGD). *Industry water-use quota of Guangdong Province DB 44/ T 1461-2014*. 2014-11-10 edn (Administration of quality and technology supervision of Guangdong Province, 2014).

13. Water authority of Shenzhen City (WASZ). *Industry water-use quota of Shenzhen City* (Water authority of Shenzhen City, 2007).

14. Industry water-use quota of Xinjiang Uygur Autonomous Regions (WRDXJ). *Industry water-use quota of Xinjiang Uygur Autonomous Regions*. 2012-3-5 edn (Water resources department of Xinjiang Uygur autonomous regions, 2012).

15. Administration of Quality and Technology Supervision of Fujian Province (AQTSFJ). *Industry water-use quota of Fujian Province DB35/T772-2007*. 2007-10-29 edn (Administration of Quality and Technology Supervision of Fujian Province, 2013).

16. Administration of Quality and Technology Supervision of Yunnan Province (AQTSYN). *Industry water-use quota of Yunnan Province DB53/T 168-2013*. 2013-9-30 edn (Administration of Quality and Technology Supervision of Yunnan Province, 2013).

17. Administration of Quality and Technology Supervision of Liaoning Province (AQTSLN). *Industry water-use quota of Liaoning Province DB21/T 1237-2015*. 2015-07-18 edn (Administration of Quality and Technology Supervision of Liaoning Province, 2015).

18. Wang, C. Y. & Hu, B. Q. On fuzzy-valued operations and fuzzy-valued fuzzy sets. *Fuzzy Sets and Systems* **268**, 72-92 (2015).

19. Statistic Bureau of Dalian City. *Dalian statistical yearbook*. 350-372 (China Statistics Press, 2012).

20. General Administration of Customs of China. *China customs statistics yearbook* (China Customs Press 2011).

21. Statistic Bureau of Dalian City. *Dalian statistical yearbook*. 323-380 (China Statistics Press, 2013).

22. Statistic Bureau of Shenyang City. *Shenyang statistical yearbook*. 63-274 (China Statistics Press, 2014).

23. Planning Research Institute for Machinery Industry (PRIMI). *The 12th Five-Year plan for equipment manufacture in Dalian City* (2010).

24. Dalian Municipal Government. *Twelfth Five-Year Plan for Economic and Social Development in Dalian City* (Dalian Municipal Government, 2011).
